# Functional engineering strategies of 3D printed implants for hard tissue replacement

**DOI:** 10.1093/rb/rbac094

**Published:** 2022-11-24

**Authors:** Cen Chen, Bo Huang, Yi Liu, Fan Liu, In-Seop Lee

**Affiliations:** College of Life Sciences and Medicine, Zhejiang Sci-Tech University, Hangzhou 310018, PR China; College of Life Sciences and Medicine, Zhejiang Sci-Tech University, Hangzhou 310018, PR China; Department of Orthodontics, School of Stomatology, China Medical University, Shenyang 110002, PR China; Department of Orthodontics, School of Stomatology, China Medical University, Shenyang 110002, PR China; College of Life Sciences and Medicine, Zhejiang Sci-Tech University, Hangzhou 310018, PR China; Institute of Human Materials, Suwon 16514, Republic of Korea

**Keywords:** hard tissue replacement, bone regeneration, 3D printing, additive manufacturing, functional engineering

## Abstract

Three-dimensional printing technology with the rapid development of printing materials are widely recognized as a promising way to fabricate bioartificial bone tissues. In consideration of the disadvantages of bone substitutes, including poor mechanical properties, lack of vascularization and insufficient osteointegration, functional modification strategies can provide multiple functions and desired characteristics of printing materials, enhance their physicochemical and biological properties in bone tissue engineering. Thus, this review focuses on the advances of functional engineering strategies for 3D printed biomaterials in hard tissue replacement. It is structured as introducing 3D printing technologies, properties of printing materials (metals, ceramics and polymers) and typical functional engineering strategies utilized in the application of bone, cartilage and joint regeneration.

## Graphical Abstract

**Figure:**
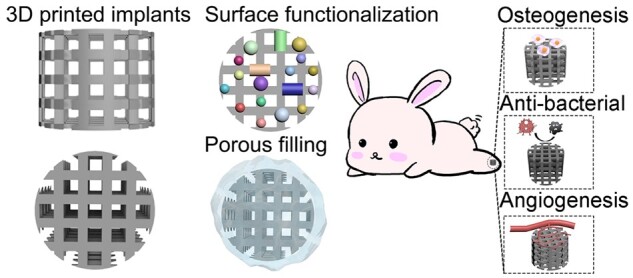

## Introduction

Bone acts as the supportive framework, protecting vital organ and maintaining multiple functions of human body. The bone disorders, referring to the structural and/or functional abnormities of bones, seriously affect patients’ physical function and mental health. Indeed, the ageing population and unhealthy life styles result in an increasing occurrence of bone defect, including fracture, osteoporosis, osteoarthritis, congenital deformity, traumatic injury and oncologic resection [1]. As a result, bone is the second most frequently transplanted tissue worldwide, with over two million maxillofacial surgeries and 2.2 million orthopedic surgeries in China every year.

The development of bone defect treatment starts from bone biology. Structurally, bone is composed of the dense and solid cortical bone, which presents highly peripheral supporting structure, and cancellous bone providing honeycomb-like network with lamellar trabecula inside [2]. The main compositions of bone are inorganic minerals, extracellular matrix (ECM) and various growth factors (GFs). Partial nanostructured ECM produced by osteoblasts provides favorable platform for cell adhesion, proliferation and differentiation. Mineralized portion of ECM undergoes the deposition of calcium-phosphates in the form of hydroxyapatite (HA) interacting with type I collagen and other non-collagenous proteins [3, 4]. Bone homeostasis depends on the dynamic metabolism of bone resorption and formation. Normally, the physiological bone remodeling ensures small bone deformities to be cured through host tissue self-regeneration. However, critical-size bone deformities need bone transplantation through surgical operation. Ideally, non-immunogenic autograft is the best choice based on bone biology mentioned above. Whereas, it requires a second operation for tissue harvest, and the limit sources become the main obstacle for large-sized defects. Although allograft is an alternative choice, immunological rejection and infection need to be carefully handled. Therefore, the development of artificial bone substitutes is essential for bone tissue engineering. Bone tissue engineering includes the utilization of biomimetic scaffolds, inducible cells and growth factors. Among them, porous scaffolds that mimic the ECM component and structure, provide mechanical supporting and biological environment for cell attachment, thereby forming new bone in defect area [5–7].

Three-dimension printing (3DP), also named as additive manufacturing (AM), was first used for fabricating models and prototypes in 1980s [8]. Compared with traditional manufacturing methods that proceed by removal of material to obtain the 3D object, AM is unique in their layer-by-layer adding and bonding material fashions to form solid 3D objects, enabling manufacturing processes that automatically produce complex structures directly from computer-aided design (CAD) models with high resolution and sophistication. These technologies are based on a layered manufacturing paradigm that builds solid objects by incremental material deposition and fusion of cross-sectional layers. By breaking down complex 3D shapes into simple 2D layers, the assembling of complex structures can be dramatically simplified under the instruction of CAD models [9–11].

In the last decades, the rapid development of AM technology has been widely recognized as a promising way to fabricate bioartificial bone tissues. Importantly, ideal bone scaffolds require various properties, including biocompatibility, mechanical integrity, bioprintability and osteoconductivity, which are mainly determined by the physical and/or chemical properties of printing materials [12]. Metals, ceramics and polymers are widely used as printing materials in bone tissue engineering [13–16]. After scaffolds implantation, the interaction of materials and surrounding tissues could directly attract cell adhesion and modulate cell behaviors including adhesion, proliferation, differentiation and apoptosis, thereby affecting biocompatibility and osteogenesis of bone scaffolds [17]. Apart from the decoration of bioactive interface above, biomimetic strategies to incorporating functional materials inspired by the chemical compositions and structures of biomolecules or their specific functionalities into implants during 3D printing process provide multi-functions and desired characteristics which can be applied in bone tissue engineering under various conditions. 3D printing scaffolds with functionalized characteristics could not only present the capacity of modulating the interaction of cells/biomolecules with scaffolds, but also obtain an optimized functionality to promote the physicochemical and biological performance of scaffolds in bone tissue engineering [18, 19].

This review covers 3D printing technologies, printed materials and functional engineering strategies for hard tissue substitutes. In detail, we provide a comprehensive overview of widely used 3D printing technologies for hard tissue regeneration. The properties of printed materials (metals, ceramics and polymers) for hard tissue implants are outlined. Additionally, typical functional engineering strategies of printed materials in bone, cartilage and joint applications are highlighted.

## 3D printing technologies for hard tissue replacement

**Figure 1.**
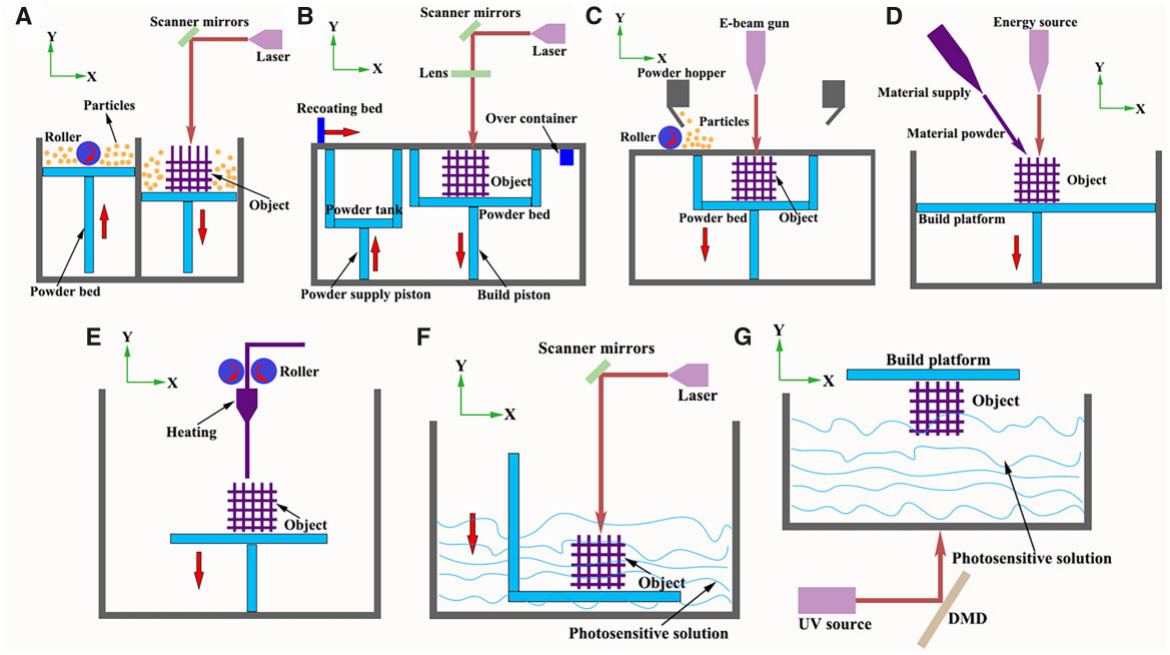
Schematic illustration of commonly used 3D printing technologies [12, 21, 32, 33, 37–39] (**A**: SLS; **B**: SLM; **C**: EBM; **D**: DED; **E**: FDM; **F**: SLA; **G**: DLP).

American Society for Testing and Materials (ASTM) has defined AM as ‘a process of joining materials to make objects from 3D model data, usually layer upon layer, as opposed to subtractive manufacturing methodologies’ [20]. Two main groups are divided for 3D printing in medical application: cell-free printing technologies and cell-laden bioprinting technologies [21, 22]. Obviously, the requirements of printing materials are different: metals, ceramics and some synthetic polymers that possess non-toxic and high stability are mostly used for cell-free printing technologies. Otherwise, cell-laden bioprinting technologies refer to living cells and materials are simultaneously printed, which had certain restrictions of printing temperatures and pressures, physical and/or chemical properties of printing materials (also known as ‘bioinks’), and cell sources [23]. According to its work principle, AM technologies are divided into powder-based systems, inkjet-based systems, materials extrusion and vat photopolymerization, whose applications are widely including metals, polymers, and ceramics (Fig. 1 and Table 1) [12, 21, 22, 24–42].

**Table 1.** Overview the 3D printing technologies

	Technology	Power source	Printing materials	Print speed	Particle size		Layer thickness		Ref.
Cell-free	DED	Laser/electron beam	Metal	Fast	25–75 μm		250 μm		[40, 41]
	SLS	Laser beam	Metal/ceramic/polymers	Slow	50–150 μm		20–100 μm		[42]
	SLM		Metal		20–60 μm				[24, 36, 39]
	EBM	Electron beam	Metal	Fast	45–105 μm		50 μm		[39]
	Binder jetting	Thermal source	Metal	Medium (6–14 mm/s)	Submicron–150 μm		15–300 μm		[25]
	FDM	Thermal	Thermoplastics; polymer-metal composites	Slow (200 µm–10 mm/s)	–		100–200 μm		[26, 35]
					**Viscosity**	**Cell viability**	**Resolution**	**Cell density**	
Cell-free/cell-laden	SLA	UV	photopolymers	Fast	None	>90%	60–150 µm	<10^8^ cells/ml	[22, 27]
	DLP	UV/visible light	photopolymers	Fast than SLA		Higher than SLA	25–50 µm		[28–31, 34]
Cell-laden	Inkjet bioprinting	Thermal/piezoelectric	Thermoplastics	Fast (1–10^4^ droplets/s)	Low (3.5–12 mPa/s)	>85%	75 µm	<10^6^ cells/ml	[9, 28]
	Extrusion bioprinting	Pneumatic/pressure	Natural/synthetic polymers	Slow (10–700 mm/s)	High (30–6 × 10^7^ mPa/s)	80–90%	200–1000 µm	>10^8^ cells/ml	[21]

### AM technologies

#### Powder-based AM

Powder-based AM technologies utilize thermal energy to selectively fuse regions of a powder bed with metallic and/or ceramic powders, which are divided into powder-bed based printing system and direct energy deposition (DED) system [38, 43].

##### Powder-bed-based printing system

The representative powder-bed fusion printing system includes selective laser sintering (SLS), selective laser melting (SLM) and electron beam melting (EBM) systems [39]. In SLS or SLM systems, powder particles with mean sizes ranging between 20 and 60 μm are placed on build platform as starting materials. Based on the presetting procedure in computer, a high-power laser as energy source scans the surface in a specific 2D pattern to sinter or melt of the powder particles [44]. After the first layer is created, the fabrication piston is lowered and a fresh layer of powder material is recoated across the top surface by a roller in powder delivery system. The resolution and surface roughness of fabricated objects by SLS are determined by particle size of utilized powders where larger particles generally cause lower spatial resolution and higher surface roughness [45]. One of the disadvantages of SLS is incomplete melting of the powder particles, leading to the porosity between the original particles, which affect density of fabricated objects. The amount of free volume is dependent on particle size distribution, printed materials and printing parameters [46, 47]. Therefore, subsequent post sintering or heating treatment is required to improve properties of fabricated object. While, on the other hand, SLS-fabricated objects are light and porous, that can be advantageous in some specific applications, for example, scaffolds require large surface areas for cell growth in tissue engineering [48, 49]. In SLM system, the material powders are not sintered but completely melted directly at processing point by laser source. Thus, SLM-fabricated parts present an improved surface quality, density and superior mechanical strength by higher laser density [50, 51]. The whole process is performed in an inert gas (i.e. argon or nitrogen) filled chamber, which minimizes the risk of oxygen and hydrogen.

EBM technology applies electron beam energy (>100 kW/cm^2^) to melt the metal powder and fabricate metallic object that can have a complex structure. The printing process undergo in a vacuum chamber, preventing the inclusion of oxygen into the system [52]. The beam controlled by electromagnetic lenses initiate heating the powder layer with a higher scan speed, followed by completely melting the powder layer based on the geometry defined by the computer design until the desired object completion. The most common metallic materials for EBM printing include titanium (Ti) alloys (Ti–6Al–4V, Ti–Al–Nb and α + β alloys), cobalt–chrome alloys (Co–Cr–Mo, Co–Ni–Cr and Co–Cr–W–Ni), stainless steel (316L) [53, 54]. Fousová *et al*. [55] focused on a comparison of SLM and EBM in terms of the mechanical properties of Ti6Al4V alloy. The internal defects of SLM resulted from insufficient melting, while spherical pores in EBM resulted from gas entrapment. More importantly, due to a higher surface roughness and more harmful defects distributed across the Ti-based samples, fatigue strength reached 115 ± 13 MPa for EBM when compared with 220 ± 24 MPa for SLM, indicating SLM seems to be a better choice for the fabrication of porous structures.

##### DED system

DED is a 3D printing process that employs focused thermal energy (i.e. laser or electron beam) to produce fully dense and functional metal implant by depositing fused metal powders. Metals in wire form are melted to form an object in a vacuum chamber. The electron beam melts up the metallic wire and thereby creates a melt pool in which more wire is fed into. A computer guides the movement on a non-stationary build platform so that a material layer is applied to those areas where the desired object needs to be built up, and the process is repeated until the whole object is built up layer by layer [56]. One major advantage of DED is the rapid manufacturing and large building capacity, when compared with powder-bed fusion. Another advantage is that the process of using metallic components with electron beams produces a high vacuum environment, which is a contamination-free work zone, without the need of additional inert gasses that are often used with laser-based processes [57]. DED allows a wide range of different metal materials including Ti, tantalum, stainless steel, aluminum alloys, nickel-based alloys and Ti aluminides. However, the shortcoming is that the accuracy of fabricated object by DED is inferior to other printing technologies. The excess materials on surface structure of fabricated object need to be removed and precisely refined through post printing process [58–60].

#### Inkjet-based AM

Inkjet-based printing systems refer to the AM process which liquid drops of fabricated materials are selectively deposited in a layer-by-layer manner.

Binder jetting, one of typical types of inkjet-based printing system, is composed of binder solution reservoir, powder reservoir and a build platform. During printing process, powders are firstly sprayed to form one powder layer, followed by jetting binder solution that acts as ‘glue’ to bond the powder particles together. Once the first layer is formed, the same procedure repeats in a layer-by-layer pattern until the desired object is completed. Compared with other printing technologies, binder jetting is compatible with virtually any powder material. Another advantage of binder jetting is that printing process occurs at room temperature and atmospheric pressure. By avoiding the use of expensive sealed chambers for vacuum, the build volume of binder jetting machines (up to 2200 × 1200 × 600 mm) is among the largest compared with all other AM technologies while still maintaining the high resolution [25]. However, in consideration of the object stability, post-processing steps (curing and densification) are required to enhance mechanical properties.

In addition to non-biological materials for binder jetting, liquid droplets incorporated of biocompatible polymers and encapsulated cells (also known as ‘bioinks’) can be printed simultaneously by thermal or piezoelectric printing nozzle [37]. In thermal bioprinters, the increasing pressure resulting from the heating force of bioink droplets to eject on built platform. However, the sizes of liquid droplets are heterogeneous, easily clogging the nozzle [61]. The heating temperature can reach at the maximum of 300°C. Nevertheless, it lasts for very short of time for ejecting process, only resulting in a slight increase of system temperature (4–10°C) [62]. Thus, encapsulated cells can still retain high viabilities. In piezoelectric bioprinters, bioink droplets are generated by piezoelectric actuators, which remain uniform size and shape of liquid droplets.

#### Materials extrusion AM

In extrusion-based AM, materials are extruded through one or multiple print heads by pneumatic pressure or mechanical force. In printing process, a continuous force allows materials to be extruded as a continuous line of ‘printing ink’, rather than liquid droplets, through one or multiple micro nozzles.

Fused deposition modeling (FDM), also named as thermoplastic extrusion (one of extrusion-based AM), is the layered deposition of molten thermoplastic materials via a heated nozzle. The filament or pellet forms of thermoplastics are heated into semi-liquid states and extruded onto platform. Most synthetic polymers, including polyurethane (PU), poly-caprolactone (PCL) and poly-lactic acid (PLA) are ideal printing materials for FDM in medical application, which can be used for customized patient-specific medical devices. The obvious advantage of FDM is quickly building construct with geometric accuracy and excellent mechanical properties. Meanwhile, bioactive substances including growth factors, antibiotics, drugs, can be incorporated into thermoplastic polymers to enhance the biological properties of fabricated objects, whereas the use of FDM is limited for living cell printing directly under the high temperature of melting [63].

Unlike FDM, extrusion bioprinting do not involve any heating process, indicating polymeric hydrogels with living cells and other bioactive substances can be extruded through nozzles by pneumatic pressure or physical force in a controllable manner. The solidification of polymeric hydrogels is achieved by physicochemical crosslinking (i.e. sol–gel transformation, polymerization and enzymatic reaction). However, the viscosities of printing hydrogels are crucial for cell viabilities. Generally, higher viscous bioinks extruded from nozzles results in higher shear-stress, which is detrimental to cell viabilities. Additionally, the printing resolution is limited, with the range between 200 and 1000 µm, compared with other technologies [64].

#### Vat photopolymerization

Vat photopolymerization is an AM process in which liquid photopolymer in a vat is selectively cured by light-activated polymerization. Stereolithography (SLA) and digital light processing (DLP) are two main lithography-based 3D bioprinting technologies.

Basis of SLA is a basin filled with liquid photopolymer, which can solidify after a certain exposure time. In printing process, liquid material is exposed by using light beam to form first layer of fabricated object on the platform. After the platform goes down by the height of one layer, the first layer is covered with photopolymer in the basin and repeated the same procedure until the desired object completion [9]. DLP follows the similar working principle as SLA. However, the light source cures the photopolymer as first layer by a digital micromirror device, which is composed of approximately 1 million micromirrors, rather than linear light beam in SLA [30, 32]. As a result, an entire layer can be cured at a time, indicating DLP exhibits a faster printing speed than SLA. Moreover, visible light can be adopted in DLP system as light source, which supports long-term cell viabilities [65]. Both SLA and DLP are safe for cell incorporation since they avoid high printing temperature and shear stress.

## Functional engineering strategies of metallic materials for hard tissue repairment

Compared with other materials, metallic materials are mostly used for hard tissue regeneration, especially for orthopedic and dental application. The requirements of implantable metallic materials include corrosion resistance, proper mechanical strength (specific strength, endurance strength and impact toughness) and high biocompatibility [66–69]. Another important issue for metallic implants is stress shielding, which could increase the risk of bone resorption and fracture [70].

### Printable metallic materials

#### Cobalt–chromium alloys

Cobalt–chromium (Co–Cr) alloys have been used for medical implants since the 1930s [71]. The chromium forms a protective Cr_2_O_3_ film when exposed to the physiological environment, presenting excellent corrosion resistant and wear resistance [72, 73]. The face-centered cubic crystal structure of Co is the predominant metal element (>60%) in Co–alloys, which is believed to be associated to high yield strength, high work-hardening rates, limited fatigue damage under cyclic stresses [74]. Cr is the primary alloying element which increases strength due to carbide formation. Co–Cr alloys are often used in permanent load-bearing implants in the orthopedic and dental fields [54, 75]. For example, Co–Cr alloys are often used for removable partial denture (RPD) frameworks [76]. Compared with conventional casting or milling methods, higher mechanical strength of dental Co–Cr alloys were achieved by using SLM technology [77]. Murr *et al.* [54] fabricated open-cellular structures of Co–29Cr–6Mo for femoral application by using EBM technology, and achieved proper Young’s modulus that matched the stiffness of knee.

#### Titanium

**Figure 2.**
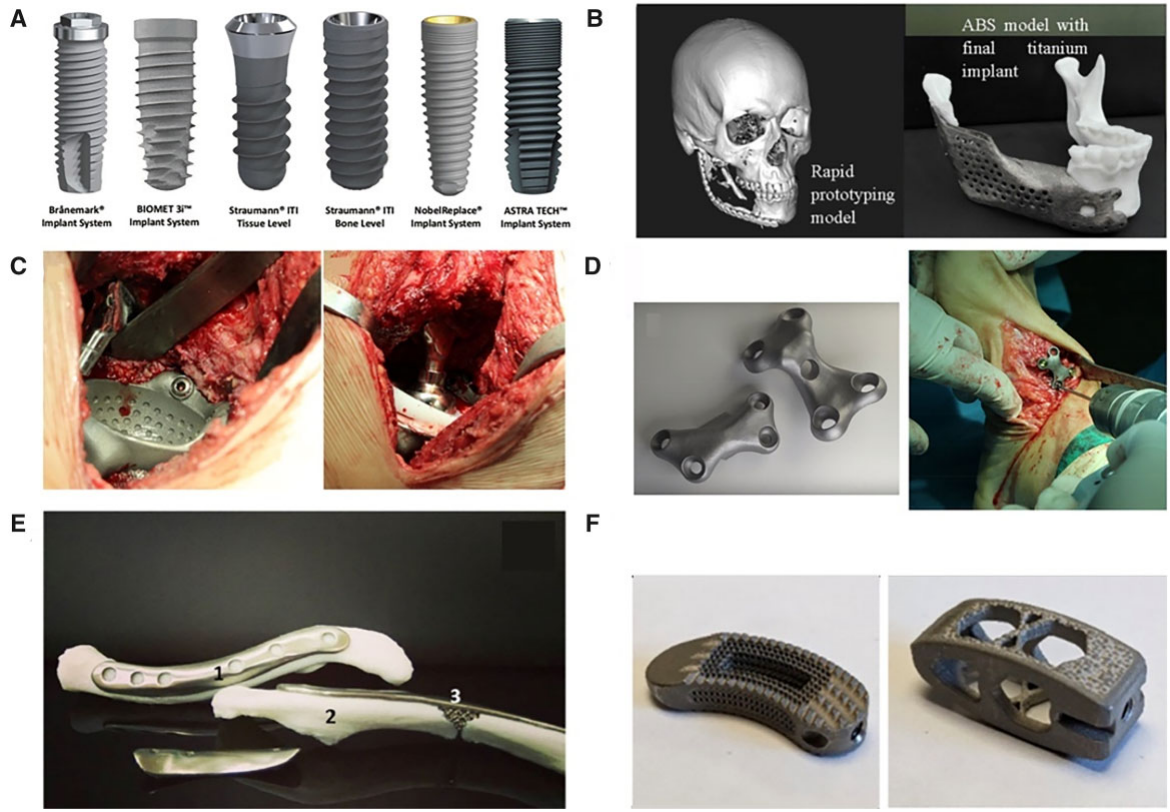
The clinical application of Ti-based implants [78–80]. (**A**: Commercially available dental implants. Reproduced with permission from Ref. [80] Copyright 2020 Japanese Society for Dental Materials and Devices. **B**: The anatomic design of the mandibular implant with a lattice structure. **C**: Hip replacement of Ti6Al4V implant using EBM technology; **D**: patient-specific Ti6Al4V implants in foot osteotomy; **E**: 3D printed Ti implant with lattice structured insert by TechMed Technion for clavicular reconstruction. Reproduced with permission from Ref. [78] Copyright 2018 Springer Nature. **F**: Activated Ti interbody cages. Reproduced with permission from Ref. [79] Copyright 2021 Springer Nature.).

Pure Ti (CP–Ti) and its alloys (Ti6Al4V) have been introduced in biomedical implants since the 1970s. They exhibit excellent physical properties as bone scaffolds, especially in load-bearing sites of bone defects, which are the most widely used materials for hard tissue replacement (Fig. 2) [78–80]. To be specific, CP–Ti is a relatively weak α-type alloy that cannot be strengthened by heat treatment, while Ti6Al4V is an (α + β)-type alloy whose mechanical strength can be increased up to 50% by heat treatment without significantly affecting its Young’s modulus. In particular, Young’s modulus of CP–Ti and Ti6Al4V is 103–107 and 114–120 GPa, which is significantly higher than that of bone tissue (7–25 GPa), easily causing stress shielding [81]. In detail, 3D printed Ti-based scaffolds could effectively avoid stress shielding, control mechanical properties to targeted implantation area and favor bone ingrowth, which widely used for hip or knee replacements, spine fusion cages, and craniofacial reconstructions [82]. While bulk Ti-based implants mostly used as dental implants with a survival rate of around 95% according to 10-year clinical observations [83–85]. The major complication is peri-implantitis, which can cause bone loss around the implant, eventually resulting in implant failure [86]. Various surface engineering strategies have been made to optimize implants’ bioactivity by increasing their surface roughness and performing physicochemical modification.

#### Tantalum

**Figure 3.**
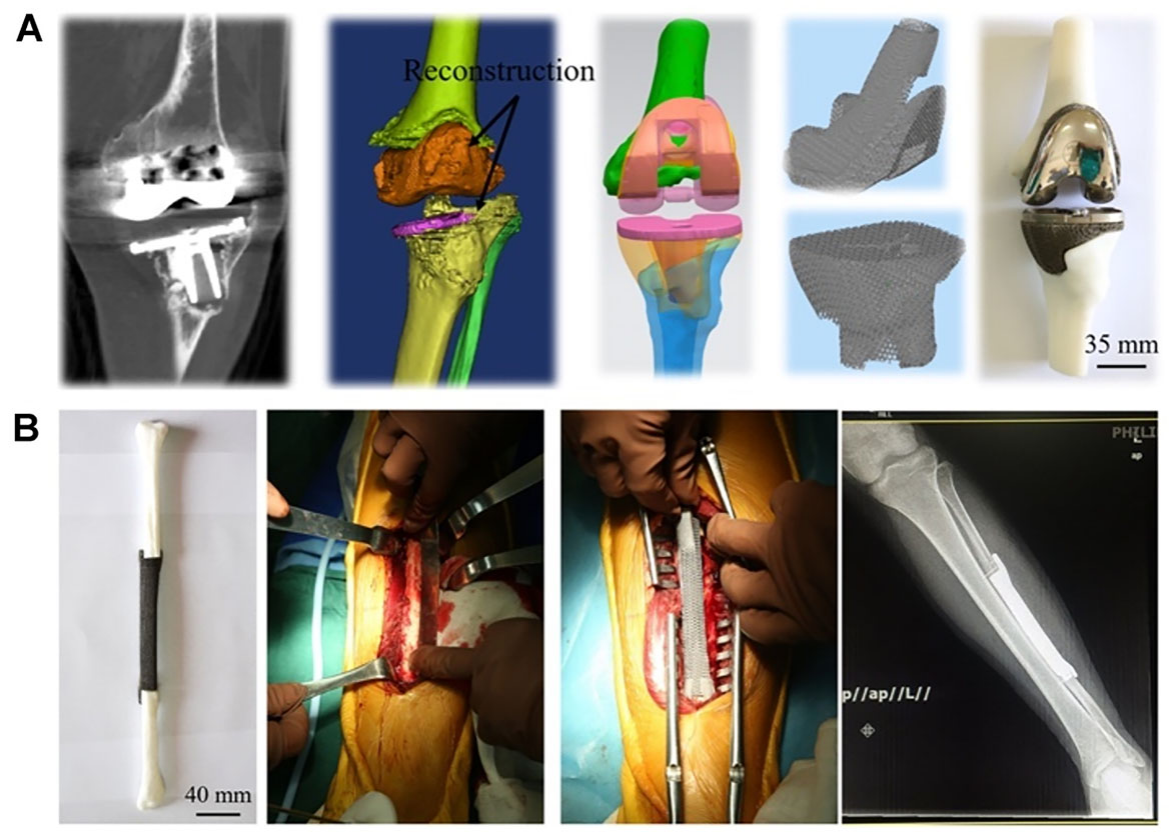
The clinical application for tantalum implants [92]. (**A**: EBM-fabricated ta lattice implants for hip reconstruction; **B**: EBM-fabricated ta fibula lattice implants.) reproduced with permission from Ref. [92] Copyright 2020 Spring Nature.

Tantalum (Ta) has been used in orthopedic surgery since the 1940s. Ta holds anti-corrosion properties *in vivo* by forming an inert oxide coating surface, which prevents electrochemical reactions and other reactions caused by metallic ions. Similar to Ti, Ta has been used as bone-substitute materials for total joint prosthesis, osteonecrosis of femoral head and dental implants [87]. However, extremely high melting temperature of Ta (3017°C), along with its high affinity towards oxygen, makes it difficult to process Ta structures via conventional processing methods [58]. Thus, SLM and EBM are commonly applied for the fabrication of Ta implants [88, 89]. Luo *et al*. [90] identified that porous tantalum scaffolds with pore size of 400–600 μm and porosity of 75% showed better performance of bone ingrowth and integration. Furthermore, some researchers verified 3D printed Ta scaffolds are superior to Ti scaffolds in resistance to compression and deformation [88, 91]. Tang *et al*. [91] achieved satisfied outcomes in 27 clinical applications (hip, fibula and femur) of EBM-fabricated Ta bone implants since 2016, indicating the potential application as bone-substitute materials (Fig. 3) [92].

More importantly, Ta is also used as coating material on account of good corrosion resistance. Several researches illustrated Ta provided high wettability and surface energy at bone-to-implant interface, facilitating favorable microenvironment for cell adhesion [92–95]. Wang *et al*. [96] provided a novel understanding on the molecular mechanism underlying the excellent biological performance of Ta coating of Ti implant under diabetic condition. Ta coating could promote the proliferation and osteogenic differentiation of osteoblast by suppressing ROS-mediated p38 pathway *in vitro*, and induced more new bones, enhancing osteointegration at bone-to-implant interfaces under diabetic sheep models.

#### Magnesium

Magnesium (Mg) is commonly known as a degradable metal for orthopedic use [97]. Therefore, the long-term side-effects of Mg implants could be minimized or avoided, and the released Mg ions may facilitate bone regeneration [98]. Another advantage is that Mg presents more comparable modulus to bone, which reduces the detrimental effects of stress shielding [99]. Somehow, it is difficult to fabricate Mg parts through 3D printing due to its highly reactive activity. The surface energy of raw Mg powders or wires increase and present a higher risk of reacting with atmospheric oxygen to enable combustion. An inert atmosphere is required to prevent exposure to oxygen. Selective laser melting, directed energy deposition and binder jetting are commonly used for the fabrication of Mg-based implants [100, 101]. Ideal Mg-based orthopedic fixators including plate, screw, pin, scaffold and ring, have strong initial mechanical strength to support the fractured bone in the early healing stages, and their proper degradation behavior match the healing process of the fractured bone [102–105].

Similar to Ti, Mg-based implants are demonstrated as clinical success especially for orthopedic bone screws. MgYReZr alloy screws (MAGNEZIX^®^) have been approved for clinical use since 2013 and extended to over 50 countries [106]. In 2015, Korea Food and Drug Administration has approved another MgCaZn screws for clinical use, in consideration of the complete healing performance of distal radius fracture after six months post-fixation [99, 107]. In China, pure Mg screws (purity: 99.99%) are widely used to repair head and neck factures with a lower rate of complication [108, 109].

### Functional engineering strategies of metallic materials

Metallic implants are most commonly used for load-bearing implant applications. The modification strategies of metallic materials including suitable physicochemical properties (i.e. fatigue and wear resistance, modulus and density) and biological properties (i.e. biocompatibility, osteoconductivity and osteoinductivity), providing functionalized bone-to-implant interfaces, which achieve long-term stabilities [110–112]. In this section, modification strategies of metallic-based implants based on post-printing treatment, physicochemical modification and surface coating are outlined (Table 2) [95, 96, 113–120].

**Table 2.** Representative examples of modification strategies on metallic implants

Substrate	Coating method	Loading material	Effects	Ref.
Ti–6Al–4V disc	Alkali-treatment; Dopamine grafting	Gentamicin (GS)/agarose	The released GS from composite hydrogel layer were able to efficiently inhibit bacterial activity.	[113]
Ti disc	Biomimetic deposition	CS		[114]
Ti mesh	Anodization	Sr/CaPs	Sr-droped CaPs/Ti mesh exhibited the higher gene expressions related to osteogenesis, and resulted in new bone formation *in vivo.*	[115]
Ti disc	Microarc oxidization	antimiR138/CS/HA	Ti with functionalized layer enhanced the osteogenesis of MSCs *in vitro*, and promoted osteointegration in rat model *in vivo*	[119]
Ti disc	Biomimetic deposition	Pac-525/PLGA microspheres/HLA	Pac@PLGA/HA coated Ti exhibited a strong cytotoxicity to both Gram-negative bacteria and Gram-positive bacteria	[118]
Ti disc	Electrophoretic deposition	Double layers: Sr/HLA; Vancomycin/CS/Gel	The drug released was more sufficient, providing a more significant anti-bacterial activity for a drug concentration of 2.74 μg.	[120]
Ti scaffold	Biomimetic deposition	BMP-loaded silica/CS	BMP-loaded silica/chitosan scaffolds exhibited the highest e bone regeneration fraction (36%) at 4 weeks	[116]
Ta scaffold	Electrostatic self-assembly	DOX/HEMA-MMA-MAA	The release of DOX from the functionalized Ta implants was up to 1 month, and inhibited the proliferation of chondrosarcoma cells	[117]
Ti–6Al–4V disc	Vacuum plasma spraying	Porous Ta	Porous Ta coating s promoted BMSCs adhesion, proliferation, osteogenesis *in vitro,* and promoted osseointegration, leading more bone formation i*n vivo*	[95]
Ti scaffolds	Chemical vapor deposition	Porous Ta	Porous Ta coating promoted osteointegration by suppressing the ROS-mediated p38 pathway under diabetic condition.	[96]

CS, chitosan; HLA, hyaluronic acid; DOX, doxorubicin; HEMA-MMA-MAA, hydroxylethyl methacrylate-methyl methacrylate-methylacrylic acid; BMSCs, bone mesenchymal stem cells.

#### Post-printing treatment

Three-dimensional printed metallic implants exhibited different microstructures with improved physical properties when compared with those fabricated by traditional casting or milling methods. However, during printing process of EBM or SLM, the recurrent melting and cooling of metal powders easily result in poor surface finish, high porosity and the formation of residual stress, which affects stability or accuracy of desired objects [121, 122]. Apart from pre-heating feedstock powder and substrate before printing for reducing the residual stress, the post-treatments after printing are necessary to improve physical properties (especially for fatigue performance) of metallic implants [123]. Post sintering or heating treatment for optimizing microstructure is required to improve properties of fabricated object. Mierzejewska *et al.* [38] demonstrated the strength and Young’s modulus of Ti6Al4V alloy fabricated by direct metal laser sintering (DMLS) before heat treatment was higher, when compared with cast and forged samples. Importantly, a decrease in hardness of DMLS samples were observed after heat treatment, indicating the significant change of microstructures. Another research by Jaber *et al.* [124] proved that the tensile strength/ductility of the Ti6Al4V alloy produced by SLM was determined by the post-heat treatment. The best mechanical properties were obtained by heat treatment at 850°C followed by cooling in the furnace, which increased ductility from 8% to 13%. The improvements in the mechanical properties after post heat-treatment are mainly due to the elimination of thermal stresses and the changes of microstructures [111, 125].

#### Physical and chemical modification

Apart from heat treatment that has been proved to especially improve mechanical properties, physical and/or chemical modification could provide macro/micro/nano-scale surface roughness that enhancing both physicochemical and biological properties of metallic implants [126].

**Figure 4.**
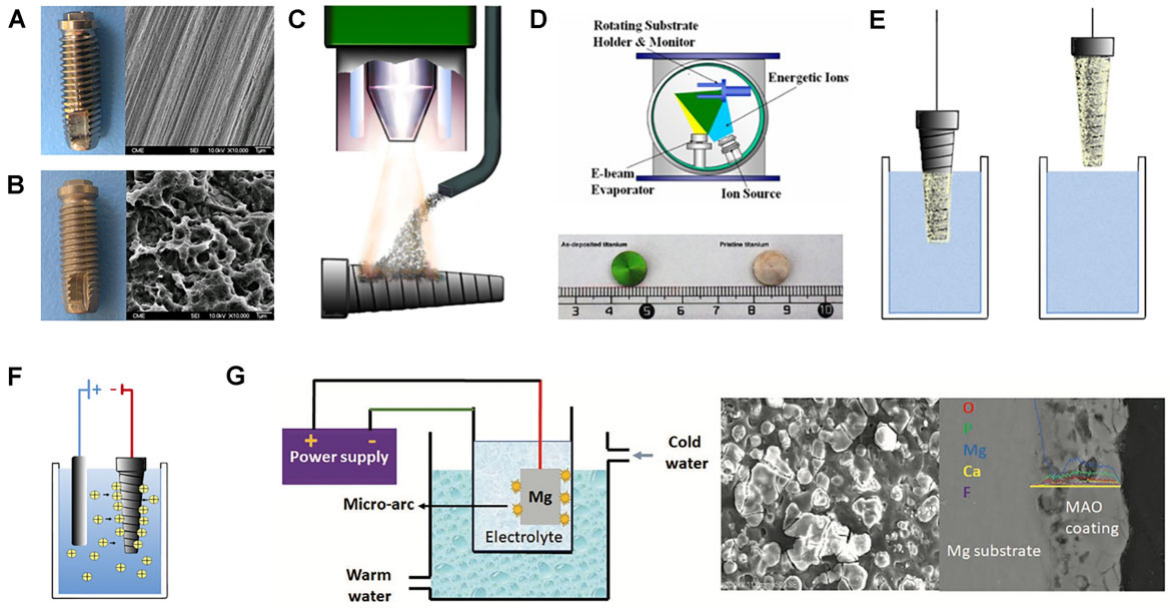
Representative examples of surface modification strategies [107, 153]. (**A**: Machined surface of Ti implant. **B**: Sand-blasted and then acid-etched implant. **C**: Plasma-spraying, to form an oxide film on the Ti surface and allows ceramic coating formation. **D**: IBAD, able to prepare bio-coatings with considerably higher adhesion strength. **E**: Dipping, sol–gel and biomimetic coating, known as simple operation and cost-effective methods. **F**: EPD, utilizing two electrodes to induce the migration of particles in solution towards the surface to be coated. Reproduced with permission from Ref. [107] Copyright 2020 John Wiley and Son. **G**: MAO treatment of Mg-based metals to form a CaPs coating. Reproduced with permission from Ref. [153] Copyright 2017 American Chemical Society).

In terms of 3D solid Ti implants (i.e. dental implants, screws and nails), mechanical modification has been used to improve surface roughness, hardness, wear resistance, corrosion resistance and wettability. Peening including shot peening, ultrasonic impact peening and laser shock peening have been widely perceived as simple and effective post-processing methods to eliminate residual stresses in the lateral direction, effectively increasing the fatigue strength of 3D printed objects [127]. Surface mechanical attrition treatment (SMAT) is one of effective methods that obtain a nanostructured layer in the treated surface of metals, which could significantly improve the friction and wear resistance of metallic materials (Fig. 4A) [122, 128, 129]. The basic principle of SMAT is the modification on surface layer of a bulk metallic materials, such as Fe, stainless steel, Ti, Co–Cr and Mg, though repeatedly impacted by multidirectional flying balls (diameter: 1–10 mm) [128, 130]. Several studies have confirmed SMAT-treated metals promoted the focal adhesion, proliferation and differentiation of MSCs [129, 131, 132].

Grit-blasting or sand-blasting/acid-etching (SLA) are commonly used physical modification methods for Ti-based materials (Fig. 4B). The macroroughness fabricated by grit-blasting is interspersed with irregularly shaped micropores while acid treatment is a simple method to provide micro/nano-structure of Ti implant [133]. Meanwhile, the acid-treated surface is usually followed by other modification strategies, including hydrothermal treatment [134]. Usually, strong acid solutions, such as HNO_3_/HF mixture or HCl/H_2_SO_4_ mixture are used to remove the oxide layer and some underlying material, as well as the contaminants on the surface of Ti implants, resulting in homogenous irregularities and increasing functional surface area and roughness [135]. TiZr alloy implants with the SLActive^®^ surface provided a long-term safe and reliable alternative to dental implants (Ti, Grade IV) [136]. The increased wettability with a micro-rough surface treated by SLA and plasma treatment could reduce M1 macrophages recruitment that were responsible for chronic inflammation, and result in the high expression level of anti-inflammatory activated macrophages (M2), which synergistically yield a microenvironment suitable to reduce healing time and enhance osseointegration [137]. In summary, compared with smooth surface, micro-topographies (surface area roughness of 1–2 μm) by SLA have been proved as an improvement in osseointegration, accelerating cell processes and shortening healing time. More importantly, metallic implants are commonly modified by sandblasting and acid-etching to reach at commercial level before clinical use.

Physical vapor deposition (PVD) and ion implantation are another two prevalent methods to improve the properties of anti-corrosion and fatigue resistance, which are expected to significantly enhance the long-term safety of Ti implants [138–140].

Besides, the activation of 3D solid metallic implants can be achieved by multiple chemical technologies including anodic oxidation, alkaline heated-treatment, plasma oxidation, which can alter the surface topography of metallic implants and benefit bone healing [141, 142]. Alkaline heated-treatment can obtain unique surface topography and provide Ti–OH sites of Ti implants, facilitating protein adsorption and reducing bacterial adhesion [143].

The increasing hydrophilicity of Ti implants, especially dental implants, can shorten the healing period by enhancing the early osteointegration while inhibiting hydrophobic bacterial adhesion [144, 145]. Usually, the implants have to be hydroxylated, rinsed under nitrogen protection, and stored in an isotonic saline solution until their use. Plasma oxidation can also be used to increase the wettability of the Ti surface [146]. Moreover, the hydrophilic surface by chemical modification has a favorable affinity to proteins and is capable to maintain the proper conformation and function of the absorbed proteins, and subsequentially encourage cell adhering onto the implant, as well as promote osteoblast differentiation [147, 148].

Micro-arc oxidation (MAO), also named as plasma electrolytic oxidation, generates a homogeneous oxide layer in the plasma state that is fabricated by applying an extremely high voltage in a suitable electrolyte (Fig. 4G) [149]. It is a well-developed method which could not only provide micro/submicron-roughness (1–20 μm) to maximize the interlocking between mineralized bone and implant surface, but also produce a porous and firmly adhesive coating on Ti surface [110, 150]. After MAO has been performed, hydrothermal treatments are usually conducted to improve its apatite-inducing ability [151]. In addition, various kinds of bioactive elements, such as Ca, P, Si and Ag, can be incorporated into fabricated layer formed by MAO to enhance the biological performance both in *vitro* and in *vivo*.

#### Surface coating

Various surface modification methods for solid metallic implants we mentioned above could provide bioactive sites on the surface that facilitate other substances to be incorporated, as well as provide multiple functions (i.e. anti-corrosion, osteogenesis, anti-bacteria and anti-inflammatory) [152, 153].

##### Calcium-based coating

In term of surface coating for metal-based implants, inorganic components, including Ca, Si, Ag or Mg, which are involved in bone metabolism and can favor bone homeostasis and augment mineralization and angiogenesis [154]. Among them, calcium-based depositions, which often refer to calcium phosphates (CaPs), are main modification strategy to form strong fixation at the bone-to-implant interface. Numerous researches have demonstrated HA layer coated on Ti implants could significantly promote osteogenic differentiation of MSCs, and induce new bone formation for bone repair *in vivo* [155, 156]. Chen *et al*. [157] gave a statement that HA coating could improve the postoperative mean Harris hip score, reduce the incidence of thigh pain and the incidence of femoral osteolysis in hip arthroplasty. More clinical studies coating demonstrated dental implants with HA coating exhibited high survival rate (97–98%) in more than 5-year follow-up studies [158, 159]. Moreover, due to the porous structures of mineral layer, bioactive molecules/antibiotics/drugs could be incorporated into CaPs layers to achieve both enhanced osteoinductivity and antibacterial effect. However, surface modification technologies are quite different between solid metal implants and porous metal scaffolds as we mention above. Therefore, we take Ti-based solid/porous implants as example, several representative Ca-based coating methods will be introduced in this section (Table 3) [160–172].

**Table 3.** Overview of bioceramic coating methods

Methods	Description	Parameter	Thickness	Advantage	Limitation	Ref.
Electrochemical deposition (ED)	Charged particles in a dispersion are migrated under electrical field towards the substrate electrode	Solution: NaH_2_PO_3_ (6 mM)/Ca (NO_3_)_2_ (10 mM) Electric current density: 20 mA/cm^2^ Temperature: 30–90°C (20 min)	50–500 μm	Simple operation; cost-effective; high deposition rate	Low adhesion strength cracking of the coating	[160, 161, 165]
Plasma spray	Molten HA particles are sprayed on the surface of metal substrate at high temperature	Plasma gas/argon gas: 50 l/min; electric current: 600 A	<20 μm	Highly crystallized HA with fine microstructure	Low adhesion strength; uncontrollable surface morphology	[160, 166]
Ion-beam assisted deposition (IBAD)	Vacuum deposition process based on ion-beam bombardment and PVD	Chamber pressure: 3 × 10^−4^ Pa Voltage of electron beam evaporator: 8.5 kV; electric current: 0.1 A	500 nm	Strong adhesion strength Thin coating	Expensive device	[167–169]
Acid/alkali-heat treatment+ Biomimetic deposition	Acids help to clean the surface of metals, followed by alkali-heat treatment to provide Ti–OH sites	HCl:H_2_SO_4_:H_2_O = 1:1:2 (30 min); 1 M NaOH (140°C, 6 h) Deposition: DPBS solution with 100 mg/l CaCl_2_ (24 h)	<30 μm	Simple operation; cost-effective; incorporation of biologically active molecules	Low adhesion strength; long time of coating process (few days)	[164, 170]
Micro-arc oxidation (MAO)	Electrochemical surface treatment technique for generating oxide coatings on metals	Electrolyte: 0.2 M C_4_H_6_CaO_4_/0.04 M C_3_H_7_Na_2_O_6_P·5H_2_O Eclectic current: 0.3 A Pulsation frequency: 800 Hz; Oxidation time: 5 min	200 µm	Homogenous oxide film layer	Microcracks	[18, 171]
Sol–gel coating	Produce almost any single- or multi-component oxide layer on metals	Preparation of HA sol: Ca/P precursors; Dip coating/spin coating	<1 µm	Low coating temperature; cost-effective; thin coating	Low wear-resistance; difficulty of porosity control	[162]
Pulsed laser deposition (PLD)	The high-power laser provides the energy source to melt, vaporize and deposit thin films	Chamber pressure: 10^−4^–10^−1^ Torr; Laser wavelength: 248 nm Pulsation frequency: 10 Hz	0.05–5 µm	Low deposition temperature Highly crystalline HA	Texturing step before coating; splashing nanoparticles on the film	[163, 172]

In term of solid Ti implants, CaPs deposition can be achieved by biomimetic precipitation, electrochemical deposition, MAO, plasma spray, sol–gel and hydrothermal deposition.

The plasma spraying deposition on metallic surface is one of physical modification methods for HA or calcium silicate coating (Fig. 4C). The molten particles are sprayed on the surface of metallic substrates at high temperature to augment their wear and corrosion resistance and bioactivity [173, 174].

Ion-beam assisted deposition (IBAD) is a vacuum deposition process based on the combination of ion-beam bombardment and PVD (Fig. 4D) [175, 176]. IBAD has the ability to prepare bio-coatings with considerably higher adhesion strength as compared with traditional coating methods. The high adhesion strength is the result of interaction between the substrate and coating atoms, assisted by ion bombardment [169, 176].

Solution-deposited biomimetic coating refers to mineral layer is nucleated and grown on the Ti substrate in solution containing Ca^2+^ (Fig. 4E). Usually, Ti substrates are firstly alkali-treated to provide Ti–O sites and immersed into Dulbecco’s phosphate-buffered saline solution under physiological conditions [177]. Biomimetic coating is a cost-effective and environment-friendly method with simple operation. Another advantage of this method is the ability to incorporate bioactive molecules that can be co-precipitated with the inorganic components [164, 172, 177, 178].

In summary, more clinical studies demonstrated dental implants with HA coating exhibited high survival rate (97–98%) in more than 5-year follow-up studies [158, 159]. Although various surface modification methods have been introduced above, only a limited number of solid implant modification technologies have been applied for clinical trials and further commercialization, while the vast majority of coatings are still in the preclinical phase.

**Figure 5.**
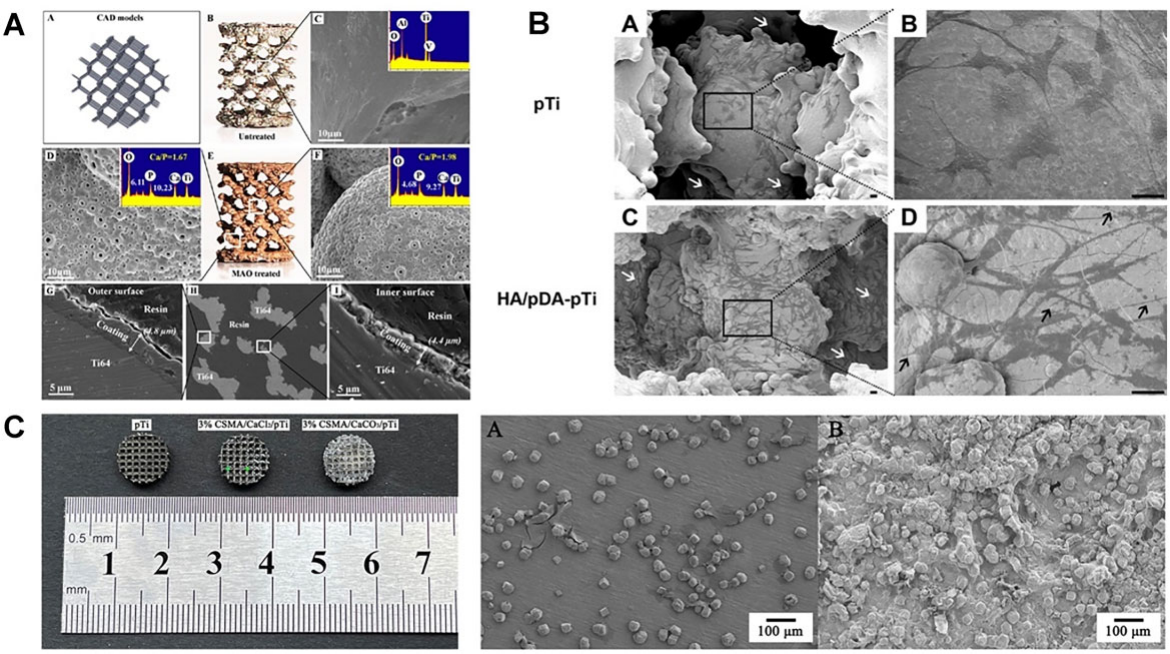
Ca-based coating for the 3D printed porous Ti-based implants (especially for internal surface) [180, 181, 183]. (**A**: the porous Ti6Al4V scaffold after MAO treatment displayed homogeneous layer of microporous titanium oxides coating containing a significant amount of Ca and P. The thickness of coating at the inner and outer surface were 4.4 and 4.8 μm. Reproduced with permission from Ref. [180] Copyright 2016 American Chemical Society. **B**: HA coating was clearly loaded on the inner surfaces of the scaffold with pre-deposited pDA film. Cells were favorably adhered on the inner surfaces (white arrows) with lamellipodia extensions (black arrow) after HA/pDA immobilization. Reproduced with permission from Ref. [181] Copyright 2020 American Chemical Society. **C**: The observation of 3D printed porous titanium filled with mineralized chitosan hydrogel. CaCO_3_ mineral layer grew inside hydrogels and wrapped up their polymer networks to provide a strong bonding between hydrogel and porous Ti scaffold.)

Unlike the solid implants, porous metallic implants (i.e. cages and scaffolds) preferred a distance osteogenesis pattern which bone grows from the periphery towards the inner scaffold [179]. Thus, the remodeling of in-growth bone needs a challenging modification strategy of internal surface. However, Common-used methods for CaPs coating including plasma spraying, IBAD deposition and electrospray deposition are not appliable for porous Ti implants, while biomimetic coating is also difficult to form uniform HA layer within internal surface of porous scaffolds. Xiu *et al.* [180] fabricated micro/nano-scale TiO_2_ coatings containing CaPs on both inner surface (4.4 μm) and outer surface (4.8 μm) of 3D printed micro-porous scaffolds, which exhibited a high efficiency in the enhancement of osteointegration of porous TiAl64 via optimizing the patterns of bone in-growth and bone/implant interlocking (Fig. 5A). Li *et al.* [181] improved biomimetic coating of HA on porous Ti6Al4V scaffolds by the introduction of polydopamine films formed by self-polymerization. The uniform HA coating on entire pore structure enhanced proliferation and osteogenic differentiation of MC3T3-E1 cells *in vitro* and improved osteointegration after implantation *in vivo* (Fig. 5B).

Apart from common-used CaPs coating, calcium carbonates (CaCO_3_) could be fabricated at the hydrogel–Ti interfaces by carbon oxide (CO_2_) diffusion [182]. The fabricated CaCO_3_ mineral layer grew inside hydrogels and wrapped up their polymer networks to provide a strong bonding between hydrogel and Ti substrate. Inspired by *in situ* mineralization method above, our group developed novel modification strategy for porous implants [183]. In detail, UV-responsive methacrylic anhydride chitosan (CSMA) was filled into 3D printed porous Ti implant, followed by the introduction of CaCl_2_ and *in situ* mineralization by CO_2_ diffusion within hydrogel, which could release Ca^2+^ continuously, resulting in promoted proliferation and osteogenesis of MSCs *in vitro* (Fig. 5C).

##### Polymeric coating

Polymeric coating layer which present highly versatile and flexible structures is another promising strategy for metallic functional modification to improve cell bioactivities [184]. Common-used fabrication methods (i.e. electrophoretic deposition (EPD), layer-by-layer deposition and electrospinning) and several representative examples are introduced in this section.

**Figure 6.**
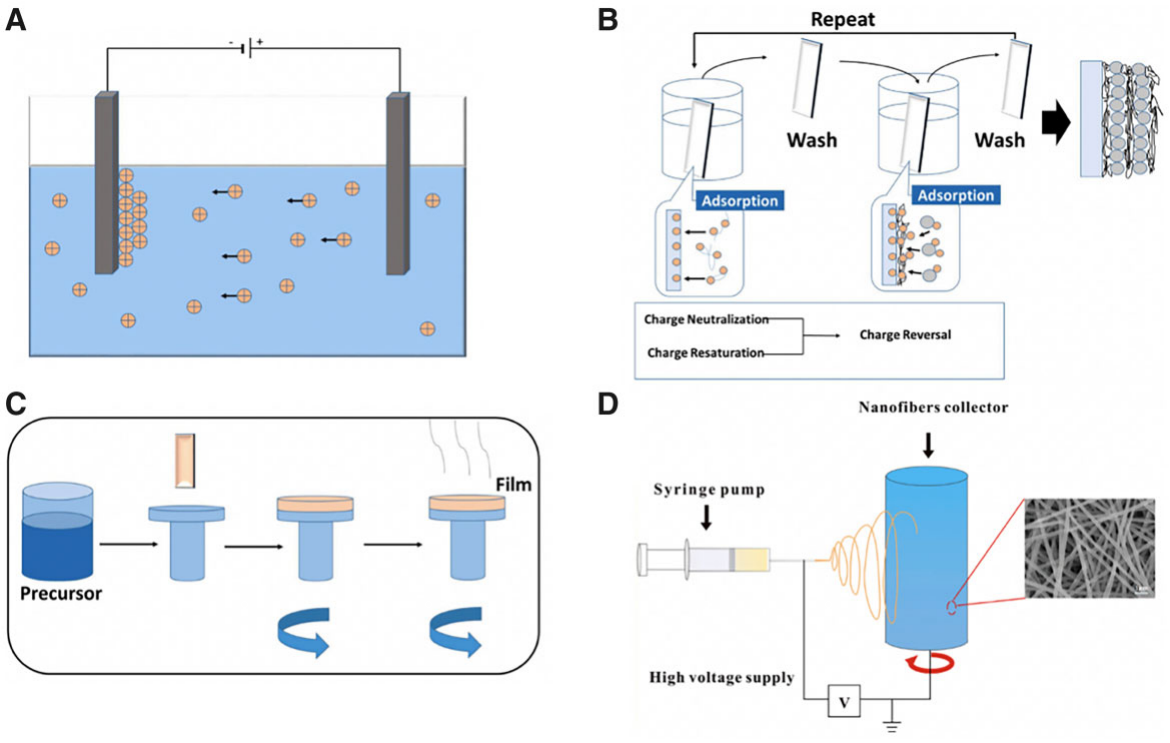
Polymeric coating strategies on metallic implants [185]. (**A**: EPD; B: layer-by-layer assembly; **C**: spinning coating; **D**: electrospinning) reproduced with permission from Ref. [185] Copyright 2021 Elsevier).

Biocompatible natural polymeric coatings are mainly inspired by the inherent structure of human bone to mimic the ECM, which significantly promote biological performance of metallic implants [118, 120]*.* Chitosan (CS), Alginate (Alg) and collagen type I (Col I) are widely used as modification materials for metallic implants due to their favorable biocompatibility, biodegradability and antibacterial properties. Compared with CaPs coating, the main effort for natural polymeric coatings is to easily load osteogenic particles or drugs along with nanoparticles (i.e. CaPs, silver, graphene oxide or zinc oxide) within orthopedic implants, which improve the bioactivity and cell attachment ability (Fig. 6) [185].

EPD is an effective method for the deposition of charged materials (i.e. chitosan) on the surface of metals. EPD is achieved by the movement of charged particles in a liquid dispersion towards the working electrode. After the deposition, a heat treatment step is normally needed to further densify the deposit and to eliminate the porosity, which could improve the mechanical properties of polymeric-based coating. The surface interactions and EPD parameters (i.e. applied voltage, deposition time and distance between electrodes) play a vital role in the coating efficiency. The cationic nature and high charge density of chitosan (CS) make it ideal for use in EPD. More importantly, CS coatings containing other polymers, nanoparticles and/or antibiotic drugs synergistically impart multifunctionality to the implant surface. Qi *et al.* [186] demonstrated gelatin nanospheres with dexamethasone (DEX) were distributed homogeneously onto chitosan-coated Ti substrate by using EPD technology, which could inhibit inflammation and stimulate osteogenesis with the help of favorable DEX release profile.

Layer-by-layer deposition (LBL) is another promising method for polymeric coating on solid substrate. The basic principle of LBL is to form integrated polyelectrolyte films (PEM) by the alteration of polymeric materials with charged sites or functional groups on their surface. The stability of the coating is determined by the static-attractive force, the strength of hydrogen bonds and covalent bonding [187]. The multilayer films could control loading and release profiles of bioactive molecules, including growth factors, drugs and DNA/RNA molecules [188–190]. Wu *et al.* [119] prepared PEMs by LBL approach with a chitosan-miRNA (CS/antimiR-138) complex and sodium hyaluronate (SH) on microarc-oxidized (MAO) Ti surfaces. The sustained release of CS/antimiR-138 from PEM-functionalized microporous Ti implant could enhance osteogenesis of MSCs *in vitro* and promoted osteointegration in rat model *in vivo*. Chitosan-decorated BSA nanoparticles (CBSA-Ns) and oxide sodium alginate (OSA) were coated on Ti scaffolds by electrostatic interaction. The synergistic effect of the hierarchical structure of assembled films and immobilized BMP2 on the scaffold improved osteogenesis of BMSCs, as well as exhibiting good antibacterial activity when incorporating vancomycin on OSA films [191]. Besides, silver nanoparticles-loaded CS-heparin PEMs were constructed on alkali-heat treated Ti substrates via LbL self-assembly technology, which promoted osteointegration and reduced microbial infection [192].

The physicochemical and biological properties of synthetic polymeric coatings can be tailored by the constituent monomers or polymers. Multiple types of synthetic polymeric coatings including poly(d,l-lactic-co-glycolic acid) (PLGA), PCL, polyvinyl alcohol (PVA) and polyethylene glycol (PEG) are frequently used as coatings for metallic implants.

Electrospinning is a promising processing technique that utilizes electrical forces to produce ultrafine polymeric fibers using polymer solutions, which can form polymeric layers (especially synthetic polymers) on solid Ti implants (Fig. 6D). Coaxial doxycycline (Doxy)-doped PCL/PVA nano-fibers were directly deposited on the Ti implant surface by using electrospinning. Doxy-doped nano-fibers coating effectively inhibited bacterial infection and enhanced osseointegration in an infected (*Staphylococcus aureus*) tibia implantation rat model [193]. Biodegradable PLGA coatings with vancomycin through electrospinning method were deposited on the surface of Ti, which exhibited a synergetic effect of anti-bacteria and osteogenesis *in vitro* and *in vivo* [194]. Lee *et al*. [195] utilized PCL as a polymer coating to improve the initial corrosion resistance of biodegradable Mg. In detail, PEO was performed to increase adhesion between the polymer and the 3D printed Mg. The improved corrosion resistance of the PCL coating resulted in the release of a lower level of magnesium ions than that in the PEO group, which can lead to r more bone formation. Stable PCL nano-fibrous layer on AZ31 magnesium alloy could also be fabricated by using electrospinning which utilizes electrical forces to produce ultrafine polymeric fibers using polymer solutions [196]. The presence of coating can be used to tailor the degradation, as well as improving cell viability, adhesion and proliferation.

## Functional engineering strategies of ceramic materials for hard tissue repairment

Ceramics, especially bioceramics, are attracted increasing attention for the application of bone regeneration due to their inherent biocompatibility and bioactivity [197–199]. Traditionally, porous ceramic scaffolds are fabricated by technologies such as freezing casting, gas forming, salt leaching and phase separation [200–203]. The development of ceramic-based printing technology overcomes the limitation of conventional methods, which mostly lack of the precise control of pore structures (i.e. pore size, porosity, inner pore connectivity) [204]. At present, the most commonly used printing technologies for ceramic includes SL, SLS, materials extrusion and BJ. As the main component and self-standing material, the printed bioceramic construct usually needs a sintering process for post-treatment to improve mechanical strength, which brings obvious difficulties to incorporate heat-liable drugs or growth factors in the printing process [205]. Due to the high-performance requirements for printing materials, only a few bioceramics are suitable for 3D printing in bone tissue engineering. Thus, representative bioceramics for 3D printing and their modification strategies as well as applications for hard tissue regeneration will be introduced in this section.

### Printable ceramic materials

#### Calcium phosphates

HA is one of most widely used bioceramics in bone tissue engineering, which is main inorganic component of human natural bone, leading to the affinity for the adhesion and proliferation of osteocytes [206, 207]. More importantly, HA can stimulate endogenous expression of osteogenic growth factors of BMSCs via multiple signal pathways [208–210]. However, the poor mechanical properties (high brittleness/low strength) and slow degradation rate of HA are main challenges in clinical translation for bone substitutes, especially for load-bearing applications [204]. Apart from HA, tricalcium phosphate (TCP) (Ca_3_(PO_4_)_2_) is another well-studied 3D printed bioceramic, with α-phase and β-phase. β-TCP has the crystal structure of a rhombohedral space group, exhibiting more stable structure and higher biodegradation rate than those of α-TCP [211, 212]. Compared with HA, β-TCP has a faster degradation rate and higher solubility. It is proved that the release of Ca^2+^ from β-TCP particles could significantly promoting osteogenesis of BMSCs *in vitro* as well as enhancing new bone formation *in vivo* [213]. Although β-TCP exhibits better bending strength and fracture toughness than HA, it is hardly used alone for load-bearing implants [213]. To solve above problems, biphasic calcium phosphate (BCP), composing HA and β-TCP in a specific ratio, is developed. To optimize the proportion, BCP scaffolds with different ratios of HA and β-TCP were prepared by ZPrinter^®^ 250 printer, the results demonstrated BCP composed of 40 wt% HA and 60 wt% exhibited favorable osteogenic differentiation of BMSCs [214]. Besides, Liu *et al*. [215] utilized DLP technology to fabricate BCP (HA:β-TCP = 4:6) scaffolds with macro-pore sizes and confirmed scaffolds with 800 mm pore size are superior for initial bone formation and maturation in a rabbit calvarial defect. Craniofacial defect, especially for intracranial hemorrhage or infarction, need precisely repair and reconstruction, otherwise, secondary infection or inappropriate substitute implantation could result abnormal swell ratio of brain tissue, which is threaten to life. Compared with Ti mesh and PEEK, bioceramic had a similar chemical composition natural bone. In clinic, 3D customized α-TCP implants were used for bone replacement in frontal-orbital region of human skull [216].

#### Calcium silicate (Ca–Si/CSi)

CaSiO_3_ (Ca–Si/CSi) is a kind of promising materials with superior sealing ability and bioactivity in bone tissue engineering [217]. It is proved that Ca–Si cement can form a HA layer on the surface of scaffolds after immersion in simulated body fluid (SBF), which are benefit for enhancing bonding between scaffold and the surrounding bone tissue [218]. Moreover, the release of Si^2+^ could up-regulate the expression level of collagen type I (CoI I), fibronectin (FN) and osteocalcin (OCN) of multiple stem cells via MAPK/ERK and MAPK/p38 signaling pathway [219]. However, the high dissolution rate of Ca–Si ceramics easily results in a high pH value, probably affects cell activities. To solve this problem, the addition of magnesium ion (Mg^2+^) is found to control the degradation rate of Ca–Si cements and improve bone regeneration [220, 221]. Another important modification strategy of Ca–Si biocaremics is bifunctional Ca–Si scaffolds with photothermal functionalization for bone tumor therapy, which will be introduced in next section.

#### Bioactive glass (BG)

BGs represent a subgroup of ceramic materials, such as silicate bioactive (45S5 Bioglass^®^), borate bioactive glass and phosphate bioactive glass. We take 45S5 Bioglass^®^ as an example, which is well studied for biomedical applications [222, 223]. Similar to Ca–Si ceramics, 45S5 glass are able to form a carbonate-substituted HA on glass surface in contact with surrounding body fluids, which is benefit for adsorption of growth factors and recruitment of osteoprogenitor cells [224]. As implantation particles or granules, 45S5 glass could effectively promote bone formation, whereas as porous scaffolds, several limitations need to be addressed [225, 226]. Firstly, crystallization of bioglass occurs close to glass transition (Tg) and impedes sintering by preventing viscous flow. Consequently, bioglass scaffolds are at least partially crystalline and often has poor mechanical properties. Another limitation is the slow degradation rate of bioglass, which cannot match the periods of bone formation [227]. BG granules also can be used as additive materials for large-scale reconstruction. Aitasalo *et al*. [228] designed patient-specific composite implant using resin matrix materials as supporting framework, which was filled with a bioactive glass. No infection or loosen of the implant was found in a 4-year follow-up, indicating the safety and osteoconductivity of bioactive glass reinforced scaffold.

Compared with conventional BG, the development of mesoporous bioglass (MBG) is attracted more attention due to the well-order mesoporous structure. The high specific surface area and large pore volume of MBG is favorable for an enhanced bone-forming ability [229, 230]. Zhang *et al*. [231] fabricated strontium-containing MBG scaffolds with controlled architecture and enhanced mechanical strength. Additionally, calcium sulfate hydrate (CSH)/MBG scaffolds were successfully fabricated using an extrusion-based printer, which had a regular and uniform macroporous structure and high porosity. CSH/MBG scaffolds could promote the adhesion, proliferation, ALP activity and osteogenesis-related gene expression of BMSCs, as well as exhibiting favorable new bone formation in calvarial defects [232].

### Functional engineering strategies of ceramic-based implants

Currently, most bioceramic materials have achieved clinical translation in the form of micro/nano particles, cements and scaffolds [204]. In term of identical bioceramic material, 3D design of a bioceramic implants offer anchoring sites to cell extensions, induce cell adhesion and further facilitate cell network formation and tissue ingrowth [233–235]. Thus, the modifications of 3D printed bioceramic scaffolds provide multiple functions, which can synergistically improve the effect of bone regeneration.

#### Structural modification

The geometrical features of porous scaffolds, including surface curvature, pore shape, pore size and porosity have great impacts on mechanical and biological performances [236–238]. Intrinsically, pore structures are highly related to mechanical behavior under variable loading conditions. Although 3D printing technologies can control identical pore size and porosity of bioceramic implants, the structural design and optimization of 3D printed ceramic scaffold is primary to solve the long-term existing issue of insufficient bone formation in the inner part of large-size scaffolds [239, 240]. Porous ceramic scaffolds are often designed with a hierarchical structure in consist of the macro-/micro- and nanostructures that facilitate bone tissue ingrowth. Generally, porous microstructures like regular strut-based structures arranged at a particular angle and pattern are used for implant and scaffold design [241]. Some specific design of structures for bioceramic implants are much more beneficial for bone regeneration.

**Figure 7.**
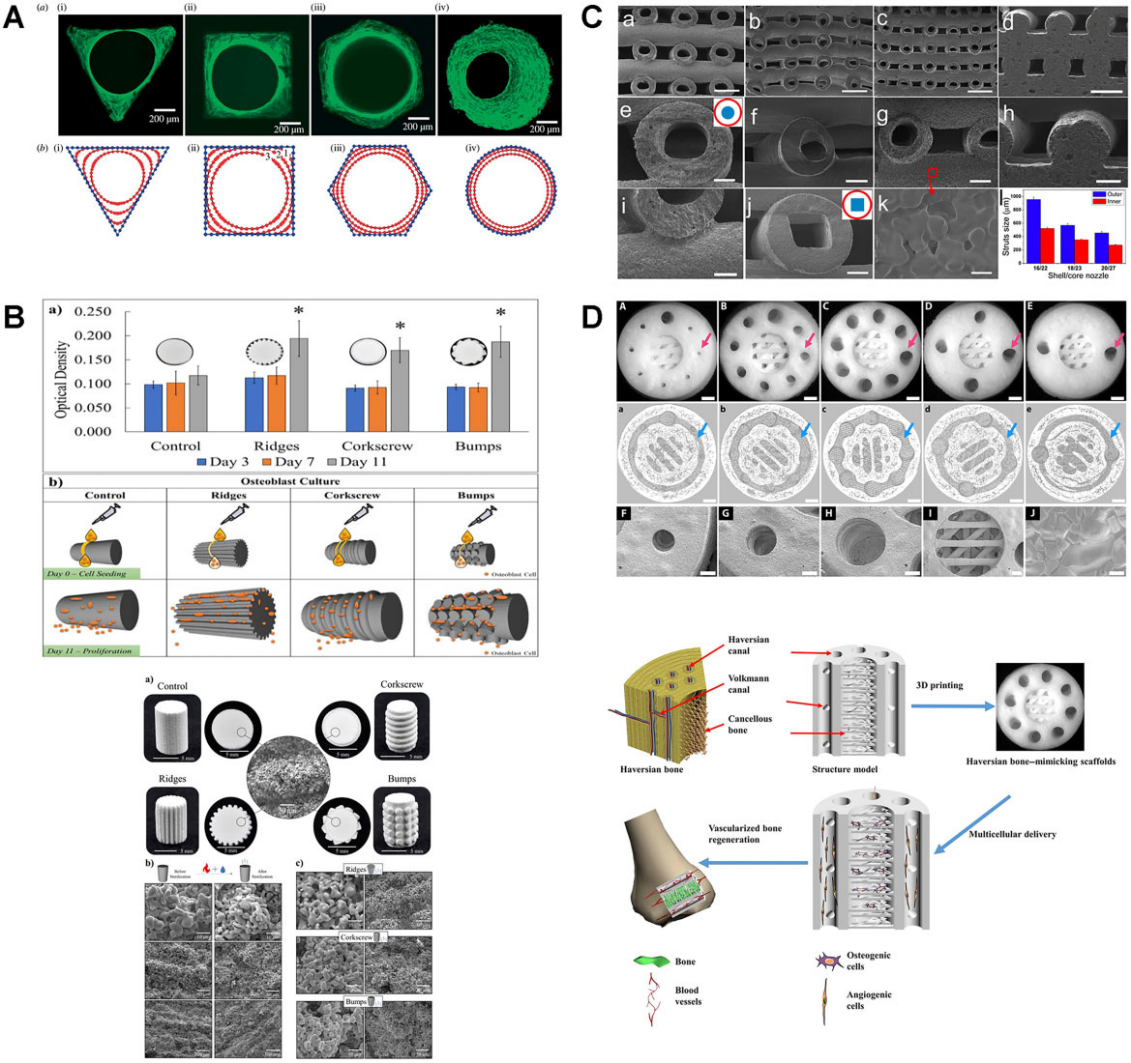
Representative examples of structural modification of bioceramics implants [243–246]. (**A**: Hydroxyapatite channels with controlled cross-sections of different geometries (triangular, square, hexagonal and circular) reproduced with permission from Ref. [243] Copyright 2008 the Royal Society. **B**: Surface topography modification via 3D printing can increase surface area to support enhanced biological response without compromising mechanical properties. Reproduced with permission from Ref. [244] Copyright 2021 Elsevier. **C**: Hollow-struts-packed (HSP) bioceramic scaffolds with designed macropores and multioriented hollow channels via a modified coaxial 3D printing strategy. Reproduced with permission from Ref. [245] Copyright 2015 American Chemical Society. **D**: A haversian bone-mimicking scaffold prepared via DLP printing reproduced with permission from Ref. [246] Copyright 2020 American Association for the advancement of science.)

Pore shape and orientation are fundamental factors relating to mechanical properties of porous implants [240, 241]. Honeycomb-pore CSi–BG scaffolds exhibited markedly higher compressive strength (∼88 MPa) than the scaffolds with other pore shapes (rectangular, parallelogram and Archimedean chord) [242]. The high strength of honeycomb pore structure was mainly attributed to its anisotropic structure and double strut wall architecture, which was widely presented in the biological systems, and had been reported to be responsible for the stability of their macroscopic architectures. Another research demonstrated that hexagonal shapes of HA scaffolds exhibited the highest compressive strength at any given porosity due to increasing contact area between printed layers, thereby resulting in a highly anisotropic architecture (Fig. 7A) [243].

In addition to mechanical properties, surface area of pore structure has an influence on cell behaviors. Bose *et al*. [244] explored topographical features with different surface area of 3D printed TCP cylindrical scaffolds, which preferably simulated personalized bone implants that could match patient’s defect site-specificity to aid bone healing (Fig. 7B). Wu *et al*. [245] prepared hollow-struts-packed (HSP) bioceramic scaffolds with designed macropores and multi-oriented hollow channels via a modified coaxial 3D printing strategy. Due to the larger surface area and higher porosity of bioceramic scaffolds with unique hollow-struts structures, such bone scaffolds significantly possessed a faster degradation rate, promoting the new bone formation in the center area (Fig. 7C).

Furthermore, inspired by the hierarchical structure and functions of bone, the haversian bone-mimicking bioceramics-based multicellular delivery system was designed and fabricated by DLP technology. Haversian canals could disperse the compressive and the flexural stress, which prevented the exceedingly early failure due to the local fracture (Fig. 7D). Also, the unique structure of haversian canals facilitated nutrition delivery, provided proper microenvironment to promote the osteogenic and angiogenic differentiation of cells, as well as enhanced both new bone and blood vessels formation *in vivo* [246].

#### External surface functionalization

In consideration of the drawbacks of bioceramic scaffolds, external surface functionalization refers to coating of bioactive layers (i.e. protein, polymers and drugs) onto 3D-printed bioceramic structure. Physical (dipping) and/or chemical immobilization (covalent deposition) are common-used methods for external surface functionalization. Physical immobilization is the simplest way to impregnate target protein onto the surface of bioceramics via weak non-covalent interactions, including electrostatic, van der Waals, hydrogen bonding and/or hydrophobic interactions. While, chemical modification is based on altering the surface energy, charge and/or functional groups [247].

Hydrothermal treatment, usually regraded as a post-printing process, is a promising modification method to form needle-shaped crystal nucleation which has a great influence on the morphological and physicochemical features of 3D-printed CaPs scaffolds [248]. Raymond *et al*. [249] reported that α-TCP scaffolds by direct ink writing presented more crystallization nanostructures, and had a major impact on permeability and protein adsorption capacity after hydrothermal processes when compared with biomimetic treatment.

Polymerization of dopamine contains a large number of bioactive groups including catechol moieties, –OH and –NH_2_, which can bind strongly to different types of materials [250]. Compared with the numerous previous investigations on pore structures of implants, Wu *et al*. [251] fabricated the nanolayers by the self-polymerization of dopamine and apatite mineralization on the strut surface of 3D printed bioceramic scaffolds by simply dropping as-prepared bioceramic scaffolds into dopamine-SBF solution for 4 days. The nanostructure of mineralized polydopamine film on 3D printed scaffolds improved osteogenesis and angiogenesis. Besides, as another biocompatible agent with high photothermal conversion efficiency.

Apart from the intrinsic osteogenic properties, bioceramics, with appropriate functionalization, are excellent candidates to promote bone and tissue regeneration along with bone cancer therapy [252, 253]. From the promises of photothermal therapy (PTT) for localized treatment of bone tumors, Yang *et al*. [254] designed a black phosphorus-reinforced BG scaffolds for the efficient localized treatment of osteosarcoma and enhanced bone formation. The significantly decreased viability of Saos-2 cells and tumor ablation effect in osteosarcoma mice model after near-infrared (NIR) irradiation, strongly demonstrated photothermal-therapeutic effects of BP-BG scaffold. More importantly, BP–BG scaffold are also favorable for biomineralization *in situ*, facilitating osteogenesis both *in vitro* and *in vivo*.

Similarly, carbon nanomaterials are recommended for their photothermal performance in bone tumor therapy [255]. Owing to the unique structure and special properties, graphene oxide (GO) processes favorable thermal conductivity and enhanced osteoinductivity [256]. Ma *et al*. [257] fabricated GO-modified β-TCP composite scaffolds combining a high photothermal effect for tumor therapy and significantly enhanced bone-forming ability by 3D printing and surface-modification strategies. Furthermore, magnetic nanoparticles (Fe_3_O_4_)-loaded GO nanolayer was prepared for the modification of 3D printed β-TCP scaffolds. The magneto-thermal property of composite scaffolds could effectively kill bone tumor cells in an alternating magnetic field, and significantly promote osteogenesis by the synergistic effect of GO and the released Fe ions.

#### Ion substitution

Differ from external surface functionalization, ion substitution usually occurs in the preparation of ceramic-based solution, and simultaneously printed as composite construct, which ensures the homogeneous distribution and well protection from degradation of bioceramic materials [225, 231, 258–260].

Ca^2+^ in HA structure can be substituted by other cations, meanwhile substitution of ${\text{PO}}_{4}^{3-}$ in the HA structure with anionic compounds has also been achieved [252]. Various researches reported that the metal ion-doped HAs stimulated osteoblast proliferation and differentiation [261]. Thus, the addition of trace elements including, molybdenum (Mo), strontium (Sr), manganese (Mn) and magnesium (Mg) into bioceramics during printing process is another important modification strategy to improve the physicochemical and biological properties of composite scaffolds [225, 259, 262, 263]. Although many literatures have reported the successful examples of ions-dropped ceramic scaffolds, conventional methods were mostly applied for these fabrications. Herein, some representative researches related to 3D printed bioceramic scaffolds with trace elements incorporation are listed as Table 4 [231, 259, 262, 264–271]. Apart from the inherent characteristics of biocreamics, additional effect including anti-bacteria, anti-tumor and anti-oxidation are achieved by some specific element nanoparticles. For example, Zhang *et al*. [270] prepared Ag modified β-TCP scaffolds through a combination of a 3D-printing method and a layer-by-layer coating technique, which could effectively kill bacteria, and had positive effects on osteogenesis by promoting the expression of an osteoblast-related gene in BMSCs. The dual effect of anti-tumor and enhanced osteogenesis can be achieved by trace elements-dropped (Cu, Fe, Mn, Co) bioactive glass-ceramic (BGC) scaffolds via 3D printing technology [271]. Under NIR irradiation, the anti-tumor investigation *in vivo* and *in vitro* found all trace elements could effectively kill tumor cells (Cu > Fe > Mn > Co), which were related to the increasing temperature and laser power density. All element-dropped BGC scaffolds could not only stimulate osteogenic differentiation of BMSCs, but also facilitate biomineralization. Among them, 3D printed Fe/BGC and Mn/BGC have great potential as bifunctional scaffolds for photothermal tumor therapy and bone regeneration. However, only a limited number of preclinical researches were performed validate the risks and benefits of 3D printed functionalized bioceramics scaffold with photothermal properties for cancer therapy. It is worth noting that the long-term and wide-spread clinical performances of photothermal bioceramics in cancer therapy need to be comprehensively investigated.

**Table 4.** Representative examples of ion substituted bioceramics in bone tissue engineering

Ceramic-based materials	Ion elements	Post-treatment	Effect	Printing parameters	Ref.
BG	Mo	1350°C, 3 h	BG scaffolds with Mo incorporation enhanced the mechanical strength, and stimulated bioactivities of BMSCs. The release of ${\text{MoO}}_{4}^{2}$- could promote chondrogenesis by activating HIF-α pathways and restrained catabolic responses by regulating TIMP3, MMP13 and ADAMTS5	Pressure: 1.5–3.0 bar Printing speed: 5 mm/s	[264]
β-TCP	Ag	1100°C, 3 h	Ag@GO nano composite-modified scaffolds were not only effective in killing bacteria, but also had positive effects on osteogenesis by promoting the expression of an osteoblast-related gene in BMSCs.	Pressure: 3 MPa Nozzle diameter: 0.52 mm Step distance: 1.11 mm	[270]
BG	Cu	1300°C, 3 h	Cu^2+^ facilitated the proliferation and maturation of chondrocytes through activating HIF pathway, and further promoting the anti-inflammatory M2 phenotype and elevating the secretion of anti-inflammatory cytokines in macrophages to reduce the damage of cartilage tissue.	Pressure: 3–6 bar Printing speed: 6 mm/s Nozzle diameter: 0.22 mm	[265]
β-TCP	Mg/Si	1250°C, 2 h	Significantly higher bone and blood vessel formation were observed for the TCP scaffolds with Mg and Si in rat distal femoral defect model.	Not report	[266]
β-TCP	Fe/Si	1250°C, 2 h	The presence of Fe and Si improved mechanical strength of β-TCP scaffolds after sintering, as well as exhibiting an enhanced early-stage osteoconduction and neovascularization *in vivo.*	pure β-TCP layers: 35 μm Doped β-TCP layers: 35 μm	[267]
BG	Cu/Fe/Mn/Co	1300°C, 3 h	Photothermal performance: Cu > Fe > Mn > Co Cu–BG, Fe–BG and Mn–BG scaffolds effectively killed tumor cells *in vitro* and significantly inhibited tumor growth *in vivo*	Not report	[271]
Ca–Si	Sr/Mg	1150°C, 45 min 1150°C, 3 h	Compared with conventional β-TCP scaffold, Sr/Mg/Ca–Si scaffolds had better apatite formation ability and bone induction performance.	Printing speed: 6 mm/s Nozzle diameter: 450 μm Filament spacing: 850 μm	[259]
MBG/AlgMC	Zn	CaCl_2_ crosslinking, 10 mins	The addition of Zn in BMG/AlgMC ink greatly reduced viscosity.	Nozzle diameter: 410 μm Printing width: 7.75 mm Strand distance: 1.8 mm	[268]
HA/MC	Sr	Glutaraldehyde crosslinking	Sr-HA scaffolds with mineralized collagen improved cell adhesion and proliferation in vitro, and exhibited more bone formation in bone defect.	Pressure: 0.25–0.5 MPa Nozzle diameter: 0.4 mm Filament spacing: 1.1 mm Printing speed: 5–8 mm/s	[262]
MBG	Sr	No	3D printed Sr-MBG scaffolds exhibit good apatite-forming bioactivity and sustained drug delivery properties, facilitating cell proliferation and differentiation	Printing speed: 9–12 mm/s Nozzle diameter: 0.4 mm Pressure: 1.5–3.8 bar	[231]
Ca–Si/PCL	Mg/Sr	No	3D-printed Mg-/Sr-doped Ca–Si-based scaffold stimulated bone regeneration via dual-stimulation of AKT and WNT signaling pathways	Pressure: 200–250 kPa	[269]

AlgMC, alginate-methylcellulose; MC, mineralized collagen.

## Functional engineering strategies of polymeric materials for hard tissue repairment

Polymers are large molecule materials made up of many smaller and identical repeating units joined together by covalent bonds [21]. Generally, 3D printed polymers can be classified into two major groups according to their original sources. The first group is natural polymers, such as gelatin, alginate, collagen, agarose, chitosan, fibrin and hyaluronic acid (HLA). The other group includes synthetic polymers, such as PEG, PU, pluronic acid and poly(lactide-co-glycolide) (PLGA). A distinction between the natural and synthetic polymers is that natural polymeric chains are full of bioactive groups, while synthetic polymeric networks are repeatable inert units (monomers) [21]. Theoretically, any polymers which have a sol–gel phase transition character can be applied in an extrusion-based bioprinting technology. In fact, very few polymers can be printed in layers at room temperature. Fortunately, polymer chains are more easily be connected to one another physically or chemically, when compared with metals and ceramics. After modification, special physical–chemical characters of polymers in response to various external stimuli, including light, temperature, pH, magnetism and electricity, which enrich the diversity of printing application. Therefore, the modification strategies of representative polymers and their applications for hard tissue replacement are highlighted in this section.

### Printable polymeric materials

#### Alginate

Alginate, an anionic polysaccharide derived from brown algae, have been frequently used as cell-laden ‘bioinks’ in 3D bioprinting processes, due to its biocompatibility, biodegradable, non-immunogenic and mild gelation characteristics [272]. However, alginate has poor mechanical properties, and uncontrollable degradation in aqueous condition. The free functional moieties, hydroxyl and carboxyl that distribute along the backbone, are benefited for alginate modification [273]. Ooi *et al*. [274] utilized thiol-ene reaction to design a modular alginate-based hydrogel system that extended the biofabrication window of alginate, allowing for printability at a lower concentration (2 wt%) with high cell survivability (>80%) in the creation of stable 3D constructs by extrusion-based bioprinting. Besides, crosslinking is another important part for alginate bioprinting. Prior to crosslinking, alginate solutions behave as non-Newtonian fluids with low viscosities that are unable to acquire a 3D geometrically defined structure. Various divalent cations (i.e. Ca^2+^, Zn^2+^, Ba^2+^) are commonly used as crosslinking agents [275, 276]. Chen *et al*. [277] evaluated the printability, physicochemical properties and osteogenic potential of four common alginate bioinks: alginate-CaCl_2_, alginate-CaSO_4_, alginate-gelatin and alginate-nanocellulose for the 3D bioprinting of accurate osteogenic grafts. Effective cell-matrix interactions were only observed in alginate–CaCl_2_ printed constructs, as well as exhibited significantly enhanced osteogenic differentiation in compared with the other three bioinks.

#### Gelatin

Gelatin is a partial hydrolyzed protein by breaking the triple helix of collagen into single-strain molecules, and widely applied for 3D printing due to its excellent biocompatibility, high water-adsorbing capacity, rapid biodegradability and non-immunogenicity [278]. Gelatin solution has a unique sol–gel transition at 28°C, which can be tuned and physically cross-linked during bioprinting by heating gelation. However, temperature-induced gelation is typically slow and unstable, which limits its long-term use in tissue engineering [37]. Additionally, the mechanical properties of gelatin need to be improved as bone scaffolds.

#### Hyaluronic acid

HLA or hyaluronan is a polysaccharide existing in living organisms composed of d-glucuronic acid and N-acetyl-d-glucosamine [279]. Like most of the natural polymers, HLA exhibits excellent biocompatibility and biodegradability, which has played an essential role in cell proliferation, angiogenesis and cell–receptor interactions [280–282]. Whereas, pure or unmodified HLA is seldomly used as printing materials alone [283]. No yield stress and shape fidelity during printing process can be achieved, due to the shear-thinning properties of HLA [283]. Thus, HLA can be employed as the supplemental agent to alter the viscosity of other polymer hydrogels. As an alternative way, HLA is the main component and self-standing material, undergoing single or multiple physical/chemical modification with shear-thinning or post-printing crosslinking for shape retention [284].

#### Chitosan

Chitosan (CS) is a cationic polysaccharide produced by partial deacetylation of chitin. It has been applied in many biomedical fields, such as bone, skin and cartilage repair, due to its low or non-toxic, antibiotic and biodegradable properties [285]. Furthermore, for drug delivery purpose, bioactive molecules encapsulated CS bioink provides the possibility to print 3D structures to enhance cell responses [286].

#### Poly(ε-caprolactone)

PCL is one of the non-hazardous polyesters obtained by ring-opening polymerization of ε-caprolactone monomers [287]. PCL exhibits slow degradation, resulting from its less frequent ester bonds per monomer. The melting point of PCL is about 60°C, while glass transition temperature (Tg) is around −60°C [22]. Therefore, it is an ideal structural material for FDM technologies. During the printing processes, PCL molecules maintain crystal states with low or moderate mechanical properties [288]. A major disadvantage of PCL is its intrinsic hydrophobicity and lack of functional groups, resulting in poor cell attachment and proliferation [289]. However, it is an excellent choice as a supporting material, especially for hard tissue replacement.

#### Polyethylene glycol

PEG is a hydrophilic, biocompatible, non-immunogenic synthetic polyether with a linear and branched structure that has been approved by the FDA as a good candidate for cell encapsulation [290, 291]. PEG hydrogels have been widely applied as fundamental materials for extrusion-based materials [291–293]. For example, a linear PEG with succinimidyl valerate end groups (PEGX) was used to crosslink a variety of polymers including GelMA, fibrinogen, and PEG amine via amino-carboxylic acid coupling. During printing process, extrudable and self-supporting bioink gels from the PEGX method yielded optimal layer-by-layer definition that enabled the ability to print thick, self-supporting constructs. After secondary post-printing crosslinking step, PEGX-gelatin bioink presented stable printed constructs with high cell viabilities [293].

#### Polyether–ether–ketone

Polyether–ether–ketone (PEEK) is a semi-crystalline synthetic polymer, as well as s a high-performance engineered thermoplastic polymer with potential to use in a variety of metal replacement applications due to its superior mechanical properties [294]. In the last decades, PEEK is considered as an ideal alternative material for Ti, which has been widely served in cranio-maxillofacial and spine fusion surgeries [295–298]. Compared with Ti (102–110 GPa), PEEK presents a much lower elastic modulus (3–4 GPa), which is close to human trabecular bone (1 GPa) [299]. The melting temperature and glass transition temperature (Tg) of PEEK are 343 and 143°C, respectively, which indicates thermoplastic PEEK can be printed by SLS and FDM technologies [94].

However, residual stress is accumulated, leading to warping and interlayer delamination during the printing process, which probably effects printing accuracy and mechanical properties [295, 300]. The investigations on mechanical properties of PEEK between 3D printing and conventional approach (injection molding) demonstrated that, FDM-printed PEEK had a wide range of tensile strength and Young’s modulus (approximately equaled or slightly exceeded to injection molded PEEK), which were influenced by printing parameters including layer thickness, printing speed, ambient temperature and nozzle temperature [301–303].

**Figure 8.**
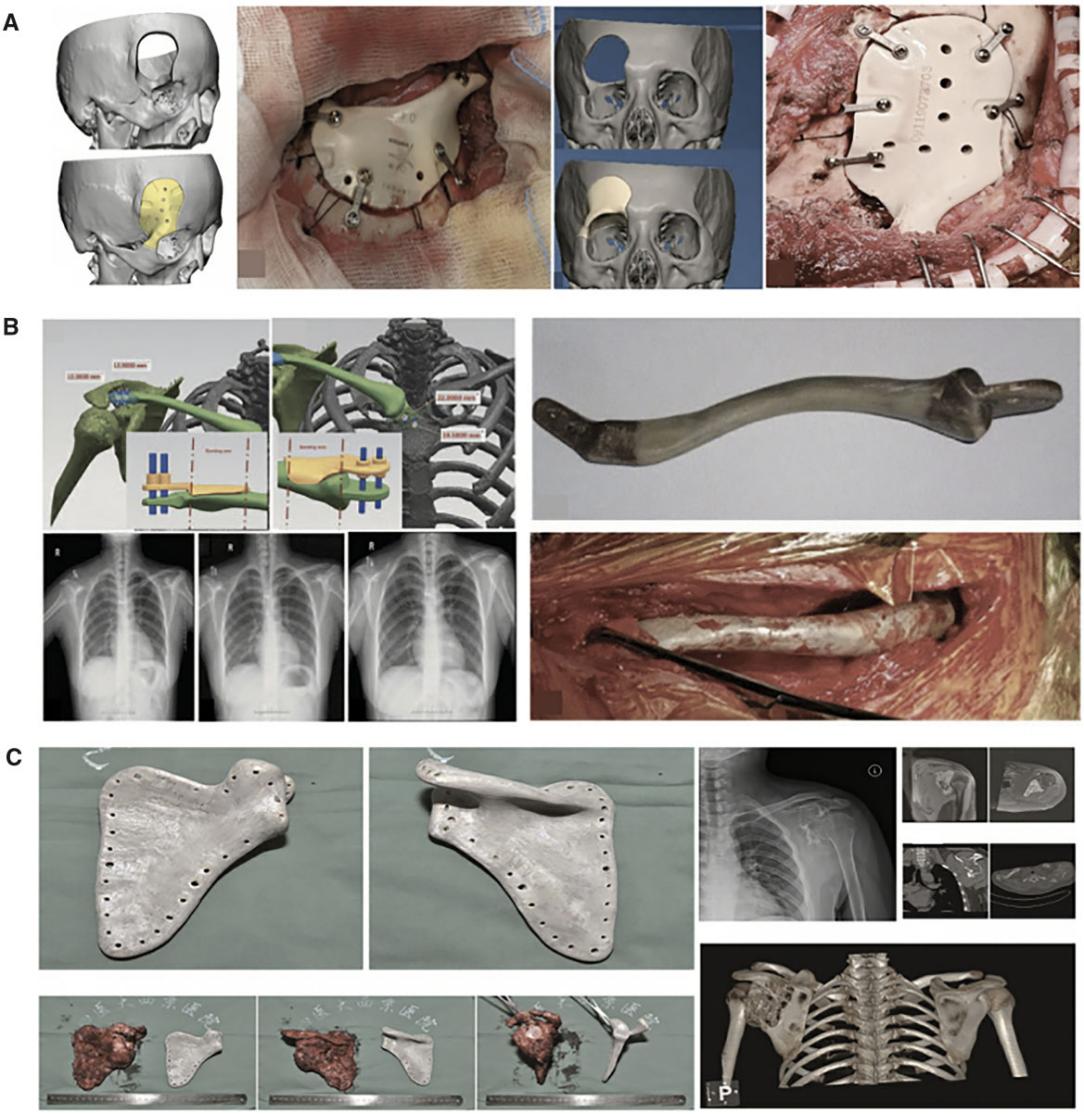
The clinical application for polymeric implants [304–306]. (**A**: 3D printed PEEK for complex craniofacial defect. Reproduced with permission from Ref. [304] Copyright 2021 Wolter’s Kluwer Health. Inc. **B**: 3D PEEK implant designed, molded and inserted into defect area in subtotal clavicle reconstruction. Reproduced with permission from Ref. [305] Copyright 2021 Wolter’s Kluwer Health. Inc. **C**: PEEK prosthesis for scapular reconstruction. PEEK prosthesis was in normal position from X-ray image after tumor resection. Reproduced with permission from Ref. [306] Copyright 2018 Elsevier.)

PEEK is a typical example for the translation in clinical use (Fig. 8). For example, patients suffering from bone-related tumors are tortured by more than tumor itself, but the tissue defects after tumor resection. Yang *et al*. [304] reported the PEEK replacement in fronto-orbital defects after the resection of fibromatous hyperplasia. The as-fabricated PEEK perfectly matched and easily embedded into the defect area to achieve individualized reconstruction in one-stage surgery. Another case was reported as reconstruction of complex bone defects in a patient suffering from chronic clavicle osteomyelitis [305]. Three-dimensional-printed PEEK prosthesis was properly fixed into the acromial end and the medullary cavity of the sternal stalk. Satisfactory cosmetic and functional outcomes were achieved after 2-year follow-up. PEEK prosthesis also can be used for the application of large joints replacement in clinic. The X-ray examination after the operation presented a good anatomical position of PEEK prosthesis, allows early functional recovery of the patient [306].

Besides, carbon-fiber-reinforced coating (CFR) is introduced for PEEK modification for clinical use. CF/PEEK is an ideal material for plates and nails due to the flexible fatigue strength and modulus of elasticity, and displayed significantly less artefacts in computed tomography (CT) and magnetic resonance imaging, allowing a precise follow-up radiograph [307]. Laux *et al*. [308] presented clinical cases with application of CF/PEEK implants in orthopedic tumor surgery. However, the failure of CFR–PEEK implants has been reported in clinic, advising the careful use of functionalized PEEK under strict supervision [309, 310].

### Functional engineering strategies of polymeric-based implants

Polymers offer greater design flexibility than metals and ceramics [311]. The properties of polymers determine their application for clinical use. In detail, natural polymers are superior to synthetic polymers in terms of biocompatibilities and often employed as cell-laden accommodations [21]. Whereas, most synthetic polymers take advantage at mechanical properties and immunogenic responses that frequently used as supporting structures [22].

#### Stimulus-responsive polymeric hydrogels

In biomedical applications, there is a great demand for ‘smart materials’ which are sensitive to external triggers or biological signals for the development of next-generation as precision medicine. Stimulus-responsive hydrogels are defined as the hydrogels with reversible or inversible phase transition *in situ* by light, heat, magnetism and/or force stimulation [312].

##### Thermo-responsive polymers

Temperature is the most frequently selected stimulus to achieve shape-transformation in bioprinted structures. Most thermo-responsive materials enable reversible deformation, and divided into two groups regarding the critical temperature, the lower critical solution temperature (LCST), and the upper critical solution temperature (UCST) [313]. Polymers are insoluble in water when possessing an UCST and become soluble below UCST [314].

Poly(N-isopropylacrylamide) (PNIPAm) is the most extensively studied thermo-responsive polymer, which is an amphiphilic polymer, possessing both hydrophilic (amide groups) and hydrophobic (isopropyl groups) chains [315]. At a temperature below LCST, PNIPAm molecules in an aqueous environment exhibit a hydrophilic behavior with an extended coil structure. When the temperature increases above the LCST (32°C), hydrophobic groups become more active, resulting in the molecules to transform into a shape resembling a compact globule [316]. Several strategies to introduce PNIPAM within natural or synthetic polymers have been developed. PNIPAm can be conjugated to hyaluronan acid (HLA-PNIPAM) as thermo-responsive HLA hydrogels that present liquid status at room temperature and gel status in the body [317–319]. HLA-PNIPAM hydrogels had great potential to support extrusion of a range of biopolymers which undergo fast gelation, thereby facilitating the printing of cell-ladened cartilage constructs [320].

##### Photo-responsive polymers

Photo-responsive biomaterials can be activated by light in a relatively wide wavelength range, including NIR, infrared (IR), ultraviolet (UV) regions and visible light [321]. Methacrylation is one of common-used methods to prepare photo-responsive polymers, including gelatin, hyaluronan acid and chitosan. Methacrylate group (MA) including glycidyl methacrylate or methacrylic anhydride can interact with carboxyl, hydroxyl and amine groups of polymers [322].

Gelatin methacryloyl (GelMA), is one typical example of photo-responsive natural polymer for 3D printing, based on a natural gelatin backbone with the introduction of methacrylate groups [323]. Hoch *et al.* [324] developed novel gelatin-based hydrogels by primary amines methacrylation and carboxylic acid moieties acetylation. After crosslinking by photo-initiator (Irgacure 2959), chondrocytes-laden hydrogels were printed by piezoelectric inkjet-based 3D printing. Almost 100% cell viabilities in hydrogels were observed after 6 h incubation. However, GelMA prepolymer solution exhibits a fast sol–gel transition at room temperature, which is a hurdle for its use in stereolithography bioprinting. Kumar *et al.* [31] modified GelMA hydrogels to exhibit slower sol–gel transition at room temperature and faster photopolymerization by optimizing the solvent, the reaction duration and the pH value. They found the modified GelMA exhibited mechanically stable structures with high resolution after DLP printing. Moreover, High cell viability and cell–matrix integration in modified GelMA hydrogels offered the versatility for a wide range of applications in tissue engineering. Although GelMA and Irgacure 2959 form prevalent system of stereolithography-based bioprinting, UV crosslinking limits penetration depth affecting the overall polymerization efficiency for large constructs. Besides, oxygen inhibition during UV irradiation results in insufficient crosslinking, which might directly impact the print fidelity of printed construct [325]. A new sight was developed by Lim *et al.* [326] who utilized visible light and ruthenium (Ru)/sodium persulfate (SPS) as visible-light initiating system to fabricate GelMA-based hydrogel constructs. To be surprised, more than 85% of cell viability was achieved in new visible light system. Meanwhile, printed constructs photopolymerized by new visible light system were completely crosslinking when compared with UV irradiation. It was indicated that optimized visible light system is more suitable for bioprinting of cell-laden constructs with high shape fidelity and cell viability.

Methacrylated HLA (HLAMA) is generated by photochemically crosslinking HLA and methacrylate using UV-light source [327]. The addition of GelMA into HLAMA exhibits tunable physical and biological properties [328]. Such hybrid printing system with two distinct hydrogel inks were developed for cartilage tissue engineering. HLAMA/GelMA provided a suitable environment for chondrogenic cell (ATDC5) encapsulation and proliferation, while cellulose nanocrystals-reinforced HLAMA/GelMA possessed excellent printability and provided adequate structural support to the 3D printed structure. Satisfyingly, the printed construct remained stable during the process of cyclic compression, as well as exhibiting persistently high cell viabilities [329].

Photopolymerization is also the common strategy to improve the strength of CS, allowing photo-responsive CS to retain its shape during the 3D printing process [330]. In term of UV-responsive CSMA, the UV curable chitosan precursor is generally mixed with photo-initiators, and exposed to UV light for crosslinking [331]. Shen *et al.* [32] utilized a LAP (Lithium phenyl-2,4,6-trimethylbenzoylphosphinate) as photo-initiator, which could be exposed to blue light (405 nm) for crosslinking. CSMA with a substitution degree (36%) can be printed into complex 3D hydrogel structures with high-resolution and high-fidelity by DLP technologies. More importantly, when compared with I2959 and UV irradiation, blue light and LAP resulted in less damage to cells.

#### Surface treatment

Similar to metallic materials, surface topography of polymers, especially synthetic polymeric materials, plays an important role to cell responses after implantation [332]. The fabricated scaffolds could be modified by physicochemical treatments. For example, the printed PCL scaffolds by Bioscaffolder^®^ device were firstly subjected to an oxygen plasma, and grafted by argon 2-amino-ethylmethacrylate (AEMA), followed by immobilizing of gelatin and physical adsorbing of FN [333]. The synergistic effect of the scaffold architecture and the biomimetic surface enabled the creation of plotted PCL scaffolds with increasing attachment, proliferation, colonization and differentiation of MC3T3-E1 cells. Park *et al*. [334] modified the surface characteristics of FDM-printed PCL/HA scaffolds using O_2_ plasma and sodium hydroxide. When compared with O_2_ plasma treatment, the alkaline treatment was favorable for exposing HA particles embedded in the scaffolds, which promoted cell proliferation and differentiation of hDPSCs. Han *et al*. [335] demonstrated post-treatment of FDM-printed PEEK including polishing and sandblasting could roughen the surface and increase hydrophilicity, facilitating cell adhesion and proliferation. A recent research by Park *et al.* [336] utilized polydopamine coating and HA layer to decorate 3D printed PCL scaffolds for BMP-2 release. The modified PCL/PDA/HA/BMP-2 scaffolds could control BMP-2 release to up to 7 days, promote cell growth, proliferation and osteogenesis *in vitro*. Besides, an injectable thermo-sensitive chitosan hydrogel with BMSCs encapsulation was incorporated into a FDM-printed PCL scaffold. The hybrid construct possessed reinforced compressive strength and favorable micro-environment for cells growth and osteogenesis via sustained release of BMP-2 for more than 1 week [337].

#### Hybrid composite hydrogels

**Figure 9.**
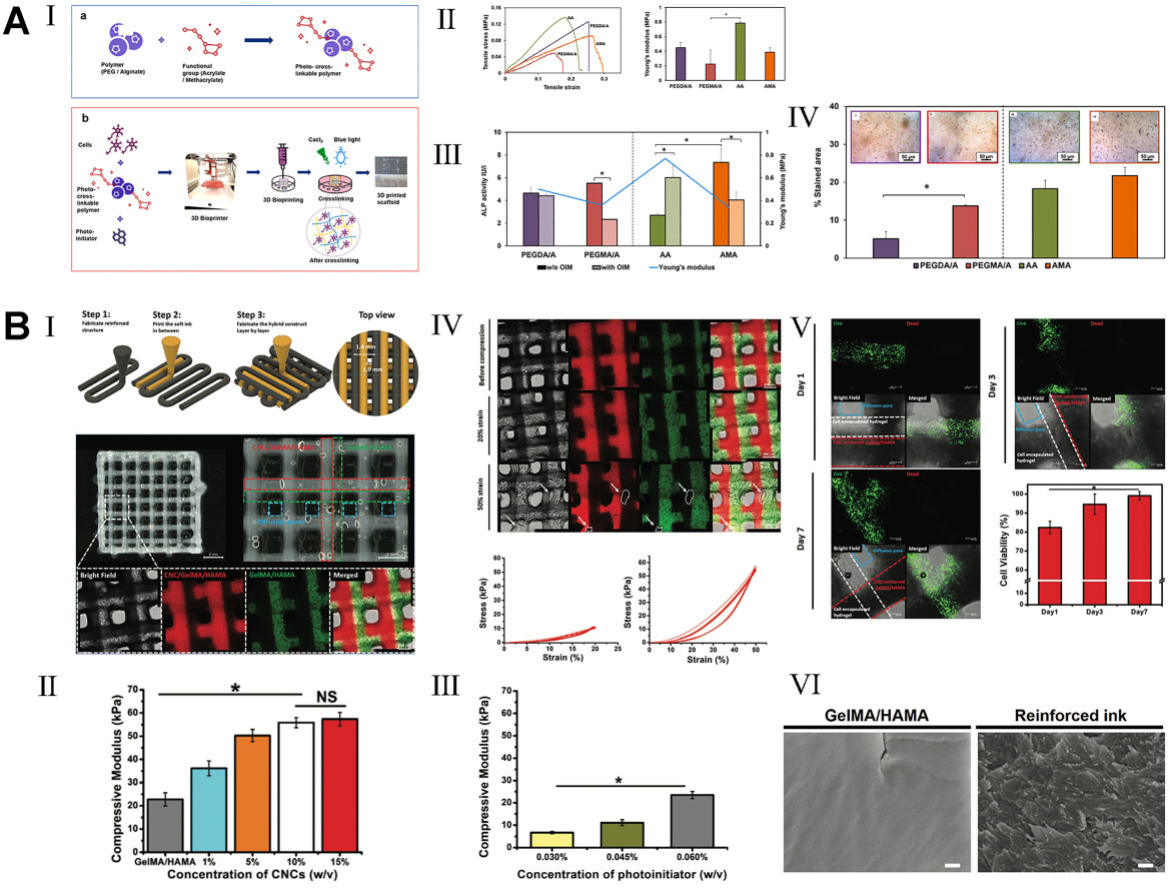
Mechanical reinforcement of composite polymers for 3D printing [329, 339]. (**A**: I. Overall 3D bioprinting process to fabricate functional PEG diacrylate (PEGDA) or PEG dimethacrylate (PEGMA)-Alg acrylate (AA) or alg ormethacrylate (AMA) crosslinked scaffolds. II. Stress-strain curve and young’s modulus (MPa) value of different hydrogels indicated the functionalized PEG and alginate gels exhibited high mechanical strength compared with natural materials. III. The ALP activities of functionalized hydrogels demonstrated the favorable osteogenic effects with any additive agents. Reproduced with permission from Ref. [339] Copyright 2021 Elsevier. **B**: I. Illustration of the hybrid printing procedure and the observation of the hybrid printed construct (the hybrid printed constructs were fabricated using the two hydrogel inks: cellulose nanocrystals (CNCs)-reinforced GelMA/HLAMA and GelMA/HLAMA inks, which were defined in the optical microscopic image by red dotted lines and green dotted lines, respectively.) II–III. Compression test for the optimal concentration of GelMA/HLAMA, reinforced hydrogel and photoinitiator. IV. Mechanical results of hybrid printed construct after cyclic compression (structural integrity was retained after 10 cycles of compression with 20% strain. After 10 cycles of compression with 50% strain, the structural defects appeared as marked by the white arrow and dot line). V. Cells remained high viability of 82.4 ± 3.3% at day 1 and reached to 99.1 ± 2.2% at day 7. VI. SEM observation of GelMA/HLAMA ink. Reproduced with permission from Ref. [329] Copyright 2020 John Wiley and Sons.)

Synthetic polymers and their composites are of great interest in orthopedic fields due to their mechanical properties that can match bone-related tissues [22]. It is noteworthy that synthetic polymers provided adequate structural support for hard tissue replacement in clinical use, especially in load-bearing sites of bone defects [338]. Thus, the incorporation of natural polymers or bioactive agents into synthetic polymers is a simple and effective way to enhance biological performance of 3D printed scaffolds (Fig. 9) [339]. Some representative examples of modification strategies on polymeric-based hydrogels are list in Table 5 [32, 284, 324, 328, 334, 335, 340–346].

**Table 5.** Representative examples of modification strategies on polymeric-based hydrogels in bone tissue engineering

Printing technologies	Bioinks	Modification strategies	Crosslinking condition	Application	Ref.
Magics EnvisionTEC (extrusion-based)	OA/Gel	Oxidization of alginate	100 mM CaCl_2_; room temperature	Not mention	[342]
Inkjet printing	Chondrocytes/GMA	Methacrylation and acetylation of gelatin	UV irradiation (Irgacure 2959)	Cartilage	[324]
Extrusion-based	Chondrocytes/GelMA/HA	Methacrylation of gelatin	UV irradiation; 37°C	Cartilage	[343]
Extrusion-based	ADA/Gel	Oxidization of alginate	BaCl_2_		[344]
Bioscaffolder dispensing system (extrusion-based)	HLAMA	Methacrylation of HA	UV irradiation Irgacure 2959	Bone	[345]
3D FDM printer (piston-based)	Ad-MeHA/CD-MeHA	Adamantane-modified MeHA; cyclodextrin-modified MeHA	UV irradiation (5 min) Irgacure 2959	Not mention	[284]
Beam projector (Stereolithography-based)	Fibroblasts/PEG/GelMA	Methacrylation of gelatin	Visible light eosin Y	Not mention	[346]
DLP	HUVECs/CSMA	Methacrylation of CS	Blue light (405 nm) LAP	Not mention	[32]
FDM	PCL/HA	O_2_ plasma and NaOH treatment	–	Bone	[334]
Stereolithography-based	Chondrocytes/GelMA/HLAMA	Methacrylation of Gel and HA	Blue light Ethyl	Cartilage	[328]
Stereolithography-based	MSC/GelMA/PEGDA	Methacrylation of gelatin acrylated PEG	UV irradiation	Cartilage	[341]
FDM	CFR/PEEK	Polish/sandblasting	Post-heat treatment	Bone	[335]
FDM	CHAp/PEEK	Not mention	Not mention	Bone	[340]

OA, oxidized alginate; GMA, methacrylated and acetylated gelatin; HLA, hyaluronic acid; ADA, alginate dialdehyde; AD, adamantane; CD, cyclodextrin; CFR, carbon fiber; CHAp, Ca_10_(OH)(PO_4_)_3_.

A linear PEG with succinimidyl valerate end groups (PEGX) was used to crosslink a variety of polymers including GelMA, fibrinogen, and PEG amine via amino-carboxylic acid coupling. During printing process, extrudable and self-supporting bioink gels via the PEGX method yield optimal layer-by-layer definition that enabled the ability to print thick, self-supporting constructs. After secondary post-printing crosslinking step, PEGX–gelatin bioink presents stable printed constructs with high cell viabilities [293]. A novel research by Piluso *et al*. [347] reported an engineered RGD modified PEG hydrogel with transient incorporation of low molecular weight gelatin (LMWG) fragments for micro-capillary extrusion-based bioprinting system. They demonstrated the incorporation of LMWG fragments enabled the micro-capillary-based extrusion of the PEG-based hydrogel with excellent shape fidelity and accuracy. Acrylated PEG (PEGDA) can be synthesized in one step by reacting acryloyl chloride with pendant hydroxyl groups [348]. Zhu *et al*. [341] prepared TGF-β1 encapsulated BSA-PLGA particles via a core-shell electro-spraying technique, and mixed with MSCs, GelMA and PEGDA as bioinks. The printed construct by tabletop stereolithography-based 3D bioprinter could significantly improve printing resolution and compressive modulus with the incorporation of PEGDA. The sustained release of TGF-β1 from composite hydrogel improved chondrogenic differentiation of encapsulated MSCs *in vitro*.

Additionally, homogeneous silicate-coated PEEK through electron beam evaporation could enhance its biological activities by promoting proliferation and osteogenic differentiation of ovariectomized (OVX) BMSCs [349]. Sulfonated PEEK was another modification strategy to enhance antibacterial and osteogenic abilities both *in vivo* and *in vitro* [350]. To be specific, Gao *et al.* [351] provided a comprehensive understanding on the immune microenvironments in the presence of PEEK with functional layers. Poly(acrylic acid) (PAA) and poly(allylamine hydrochloride) (PAH) films with tunable nanoscale porosity were prepared by self-assembly technology under two pH values. The functional films inhibited the acute inflammatory response of macrophages by down-regulating the expression of integrin and adhesion complexes, and created a favorable microenvironment for osteogenic differentiation of BMSCs, as well as promoted bone formation ability in bone defect models [352–354].

**Figure 10.**
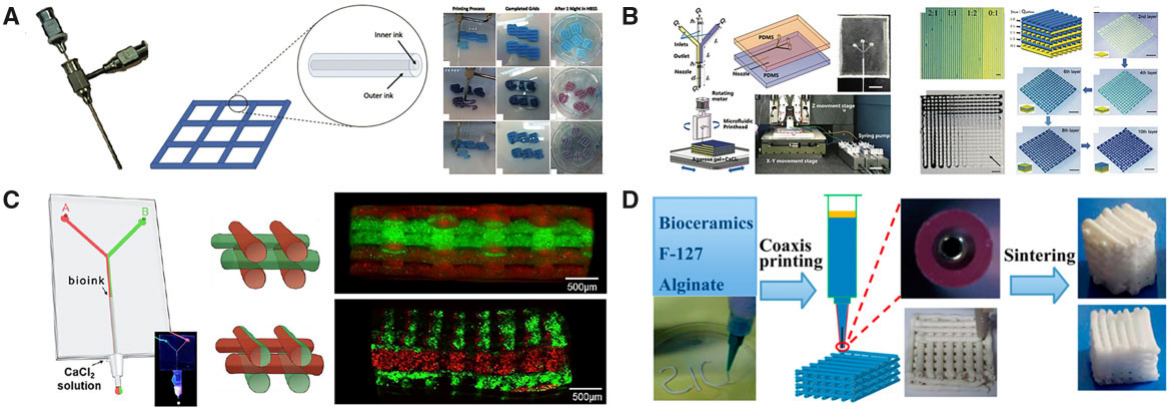
Multi-material bioprinting based on microextrusion technologies with multiple printing-heads [245, 357–359]. (**A**: Coaxial nozzles were design for gird construct with inner/outer layers. Reproduced with permission from Ref. [357] Copyright 2020 Wolters Kluwer. **B**: The design of microfluidic printheads with multiple inlets and an outlet, and the color-coded heterogenous construct. Reproduce with the permission from Ref. [358] Copyright Whioce Publishing. **C**: Microfluidic system (‘Y’ shaped channel) was used to flow two separate bioinks containing red and green fluorescent beads that through a single extruder. Reproduced with permission from Ref. [359] Copyright 2015 John Wiley and Sons. **D**: Bioceramic powders with F-127 solution and alginate were printed by extrusion printer with coaxial printing nozzle, followed by sintering to achieve mechanically stable construct. Reproduced with permission from Ref. [245] Copyright 2015 American Chemical Society.)

More importantly, hierarchical fabrications of complex construct are recently achieved by multi-material 3D bioprinters, which makes it possible to simultaneously extruding various bioinks with precise printing resolution (Fig. 10). For example, cell-laden natural hydrogels can be printed from one nozzle, and ceramics or synthetic polymers are printed from another nozzle to reinforce the structure stability. Naren *et al*. [360] designed a core-shell structure by a modified bioprinter. Ceramic paste of high viscosity could be extruded from metallic core nozzle, while shell structure consisted of cell-laden alginate hydrogels with low viscosities which were printed through an outer nozzle. After crosslinking by CaCl_2_, the alginate/bioceramic core/shell-structured 3D scaffold enhanced the mechanical properties and maintained high viability for a long culture period. Additionally, multifunctional 3D bioprinting platform with two kinds of bioprinters were designed to construct hierarchical parts [361]. Cui *et al*. [356] utilized biodegradable polylactide (PLA) fibers and cell-laden GelMA hydrogels through a multifunctional 3D bioprinting platform consisted of a FDM 3D bioprinter and a SLA 3D bioprinter to explore complex vascularized bone constructs.

## Challenges and perspectives

As the osteogenesis of 3D printed implants is a primary concern for hard tissue replacement, the implants with multiple functions are one of the most effective strategies to facilitate the osteointegration. In this review, the typical functional engineering strategies based on the properties of printed materials (metals, ceramics and polymers) are highlighted.

In hard tissue engineering, the customized implants by 3D printing technology offers precise control and high accuracy of microarchitecture. Currently, some challenges of 3D printed implants still need to be carefully addressed: (i) accuracy control of 3D printed implants based on natural bone structure; (ii) the mechanical strength of 3D printed implants matching the clinical requirements of implants under various conditions; and (iii) 3D printed implants for repairing large bone defects under various clinical and/or pathological conditions.

Due to the inherent bioinert properties, 3D printed metals mostly need bioactive surface structures after printing, no matter for solid or porous implants. Thus, physical–chemical modification methods are usually applied for the alteration of implant surface, including surface roughness, wettability and hardness. Moreover, biocompatible coating (CaPs and polymeric coating) on metallic implants can provide biomimetic microenvironments for cell responses (i.e. adhesion, proliferation, differentiation) after implantation. Differ from bioinert metals, ceramics themselves present favorable osteogenic capability. The functional engineering strategies of 3D printed ceramics implant mostly focused on the design of biomimetic structure as well as providing multiple functions (i.e. photothermal, osteogenic, antibacterial), synergistically improving the effect of bone regeneration. The properties of polymers depend on the structure of monomers that is smallest repeating unit in the polymer chains and their connections. Thus, polymeric implants are mostly undergoing chemical modification for favorable osteogenesis. Besides, in consideration of different advantages of polymers, the simultaneous or sequential printing of synthetic and natural polymers via multiple bioprinters is a potential method for fabricating multiple-functional implants for hard tissue replacement.

However, multiple functionalized implants still remain some challenges. For example, the efficiency of new bone formation is related to the degree of vascularization, especially for repairing large bone defects. Thus, enhanced angiogenesis is a major concern in cell-laden bioprinting. Antibacterial property is another strategy for researchers to functionalize 3D printed implants. Besides, further development might focus on the drug load/release profile of implants, which are released via an internal or external stimulus in a controllable manner. Although a great number of researches achieved promising results, the fabrication of multiple-functional tissue engineered scaffold is a hindrance, and further clinical translation is still at the primary stage. From general point of view, we expect the next generation of biomaterials with interdisciplinary cooperation and technological innovation will perfectly match bone defects and meet the clinical individualization requirements.
